# A systematic review and meta-analysis of the prevalence of common mental disorders in people with non-communicable diseases in Bangladesh, India, and Pakistan

**DOI:** 10.7189/jogh.09.020417

**Published:** 2019-12

**Authors:** Eleonora P Uphoff, Louise Newbould, Ian Walker, Nabila Ashraf, Santosh Chaturvedi, Arun Kandasamy, Papiya Mazumdar, Nick Meader, Aliya Naheed, Rusham Rana, Jerome Wright, Judy M Wright, Najma Siddiqi, Rachel Churchill, on behalf of the NIHR Global Health Research Group – IMPACT

**Affiliations:** 1 Cochrane Common Mental Disorders, Centre for Reviews and Dissemination, University of York, York, UK; 2 Mental Health and Addictions Research Group, Department of Health Sciences, University of York, York, UK; 3 Global Public Health Division, Public Health England, London, UK; 4 International Centre for Diarrhoeal Disease Research, Dhaka, Bangladesh; 5 Department of Mental Health Education, National Institute of Mental Health and Neurosciences, Bangalore, India; 6 Centre for Addiction Medicine, National Institute of Mental Health and Neurosciences, Bangalore, India; 7 Centre for Reviews and Dissemination, University of York, York, UK; 8 Institute of Psychiatry, Benazir Bhutto Hospital, Rawalpindi, Pakistan; 9 Leeds Institute of Health Sciences, University of Leeds, Leeds, UK; * Joint senior authorship

## Abstract

**Background:**

The prevalence of mental and physical comorbidities is unknown in South Asia, as estimates of mental ill health in patients with non-communicable diseases (NCDs) have predominantly come from studies based in the United States, Europe and Australasia. This systematic review and meta-analysis summarises evidence and provides pooled estimates of the prevalence of common mental disorders in adults with non-communicable diseases in South Asia.

**Methods:**

We included prevalence studies of depression and anxiety in adults with diabetes, cancer, cardiovascular disease, and chronic respiratory conditions in Bangladesh, India, and Pakistan, published from 1990 onwards in international and country-specific databases.

**Results:**

Out of 96 included studies, 83 provided data for random effects meta-analyses. The pooled prevalence of depression was 44% (95% confidence interval (CI) = 26 to 62) for patients with COPD, 40% (95% CI = 34 to 45) for diabetes, 39% (95% CI = 23 to 56) for stroke, 38% (95% CI = 32 to 45) for hypertension, and 37% (95% CI = 30 to 45) for cancer. The pooled prevalence of anxiety based on 28 studies was 29% (95% CI = 22 to 36). Many quality issues were identified in a critical appraisal of included studies, mostly relating to the sampling frame and selection process, the description of the methods and basic data, and the description of non-responders.

**Conclusions:**

Depression and anxiety are prevalent and underdiagnosed in people with physical comorbidities in Bangladesh, India, and Pakistan.

## Common mental disorders and non-communicable diseases

The WHO estimated that 4.4% of the global population was living with depression and 3.6% was suffering from an anxiety disorder in 2015 [1]. In low- and middle-income countries, the burden of disease caused by common mental disorders (CMDs), such as depression and anxiety, and non-communicable diseases (NCDs), such as diabetes and COPD, is high [1]. While the burden of some NCDs such as cancer and diabetes is on the rise, trends in the global burden of mental illness vary between world regions and types of disorders. In analyses with data from 1990, 2007, and 2017, depressive and anxiety disorders consistently occupied the top twenty of leading causes of the global burden of disease for men and women [2]. The prevalence of CMDs is higher among people with a physical NCD, than among those without. In the WHO World Health Surveys, between 9 and 23% of participants with one or more chronic physical conditions had comorbid depression [3]. In low- and middle-income countries, resources and access to health care for CMDs remain limited [4]. As a result, patients with comorbid physical and mental ill health are less likely to be identified, diagnosed, and treated [5].

## Integrated mental health as a global priority

There is now global consensus that the high burden of mental ill health combined with lack of access to high quality mental health services in low- and middle-income countries should be a priority for sustainable development [6]. NCDs are included in the Sustainable Development goals, and the Lancet Commission on Global Mental Health and Sustainable Development has put the integration of mental health services at the very top of the global policy agenda [7].

Building on the momentum created to address these challenges, the IMPACT project (Improving Mental and Physical Health Together, https://www.york.ac.uk/healthsciences/research/mental-health/projects/impact/) aims to improve the mental health of people with physical conditions, and the physical health of people with mental ill health, in South Asia. By undertaking a rapid evidence review of the burden of physical and mental health comorbidities in Bangladesh, India, and Pakistan, we will inform the development, implementation, and evaluation of interventions within the IMPACT project.

## Focus on Bangladesh, India, and Pakistan

In 2017, people living in Bangladesh, India, and Pakistan comprised more than 22% of the world’s population [8]. A growing number of people in these countries are living with NCDs. Data from India show that major risk factors, such as high systolic blood pressure, high fasting plasma glucose, high total cholesterol, and high body-mass index, have become more prevalent [9].

Of the total number of years of life lost globally due to premature mortality and disability because of chronic respiratory conditions, 32% of Disability Adjusted Life Years (DALYs) were lost in India [10]. Ischemic heart disease is the leading cause of lost DALYs in Pakistan, India, and Bangladesh, stroke is in the top 10 leading causes in all three countries, and chronic obstructive pulmonary disease (COPD) is in the top five in India and Bangladesh [11]. More than 18% of men and 15% of women in India over the age of 60 were living with diabetes in 2016 [12]. Many more live with poorly controlled and undiagnosed diabetes, with one study estimating that around 32 million people live with undiagnosed diabetes in India [13]. This increases the risk of developing diabetic complications, as well as the risk of developing other NCDs which often co-exist with diabetes, such as coronary artery disease [14].

In addition to the large growing burden of NCDs, the WHO estimates that 27% of all people with depressive disorders and 23% of people with anxiety disorders live in South-East Asia, making this an important region to focus efforts on the integration of mental health services into systems for treatment of NCDs [1]. The prevalence of mental and physical comorbidities however is unknown in these countries, as estimates of mental ill health in patients with NCDs have predominantly come from studies based in the United States, Europe and Australasia.

## Aim and objectives

The aim of this systematic review and meta-analysis is to summarise the evidence and provide pooled estimates of the prevalence of CMDs in adults with NCDs in Bangladesh, India, and Pakistan, to inform the development, implementation, and evaluation of interventions within the IMPACT project. To achieve this aim, we have three objectives. First, we will identify evidence and meta-analyse available data from studies exploring the prevalence of CMDs in adults with NCDs in Bangladesh, India, and Pakistan. Second, we plan to summarise evidence on determinants of CMDs in adults with NCDs, and third, we aim to explore variation in the prevalence of CMDs in adults with NCDs in Bangladesh, India, and Pakistan. We will focus this review on prevalence rates of depression and anxiety, representing the two most prevalent CMDs worldwide. We will consider NCDs in the following broad categories of illness: type II diabetes, cancer, cardiovascular disease, and chronic respiratory disease.

## METHODS

This systematic review and meta-analysis follows the Centre for Reviews and Dissemination Handbook and the guidance for reporting of Meta-analyses Of Observational Studies in Epidemiology (MOOSE) [15,16]. The PROSPERO database contains a brief version of the protocol (http://www.crd.york.ac.uk/PROSPERO/display_record.php?ID=CRD42018106502) and the full protocol is available from the study website (https://www.york.ac.uk/healthsciences/research/mental-health/projects/impact/impact-outputs/).

### Data sources and search strategy

A senior information specialist (JuW) conducted test searches to identify appropriate global health and Asian country-specific databases and web resources for relevant evidence. We searched the following databases and web resources during July to October 2018: BRAC Research & Publication website, Cochrane Database of Systematic Reviews (Wiley), Database of Abstracts of Reviews of Effect (Wiley), Global Health (Ovid), Global Index Medicus (World Health Organization) Libraries, Health Technology Assessment Database (Wiley), IndMED (ICMR-NIC), Ovid MEDLINE(R) (including In-Process & Other Non-Indexed Citations and Epub Ahead of Print), PakMediNet (PakCyber), PsycINFO (Ovid), and World Bank Group Research and Publications: Documents and Reports website.

The information specialist identified search terms and developed strategies through iterative testing of terms identified by the project team members and aided by known relevant papers. The search strategies were peer-reviewed by a second information specialist. MeSH and free text word synonyms were searched the concepts ‘common mental disorders’ (including depression and anxiety disorders), ‘non-communicable diseases’ (cancer, diabetes, cardiovascular disease and COPD), ‘prevalence studies’ and ‘South Asia’. See Appendix 1 in **Online Supplementary Document** for full search strategies including resource coverage dates where available.

We limited searches to studies published from 1990 onwards to ensure relevance to the current prevalence and determinants of CMDs.

We conducted this review within a short timeframe to inform research priorities at the start of the international IMPACT project. It was therefore not feasible to scan references of included studies, or to obtain unavailable papers.

### Study selection

We included any observational studies that were published in any language, conducted in Bangladesh, India, or Pakistan, and assessed prevalence or determinants of CMD in adults with an NCD. Studies with the following criteria were included: observational studies including cross-sectional, case-control, and cohort studies, data collected in Bangladesh, India, or Pakistan, participants at least 18 years old, one of four key NCDs: cardiovascular disease (including stroke, hypertension, angina), diabetes (type II), cancer, and chronic respiratory disease, reporting on prevalence and/or determinants of one or multiple CMDs (depression and/or anxiety disorders), and identification of patients with CMD via clinical diagnosis, use of a recognised psychiatric diagnostic instrument (eg, SCID, CIDI), or through a validated screening tool (eg, PHQ-9, GAD-7, HADS).

We excluded studies meeting the following exclusion criteria: study designs other than observational, such as literature reviews, opinion papers, qualitative studies, posters and letters, studies with a sample size of less than 20 participants, data collected in multiple countries, without the possibility to extract country-specific information on Bangladesh, India, and/or Pakistan from the paper, studies with participants under 18 years of age, for which the review authors could not extract data for adults separately, studies reporting on the prevalence or determinants of CMD without reporting the use of a recognised diagnostic instrument or screening tool, and full-text papers not available within the review period.

### Screening strategy

We stored and screened references in Mendeley reference management software (Elsevier, Amsterdam, the Netherlands, v 1.18). After removing duplicates, three researchers (EU, LN, IW) together screened titles and abstracts independently in duplicate, and disagreements were resolved through discussion, where required involving a third researcher. Two researchers independently screened all full-text papers (between EU, LN, and IW).

### Data extraction

After piloting the data extraction form, reviewers (EU, LN) extracted the following data in Microsoft Excel 2016 (Microsoft Inc, Seattle WA, USA): first author, year published, study objective, study population country (+region/ city), type of study, data source, participants, setting, sample size, CMD, CMD diagnostic criteria, NCD, NCD diagnostic criteria, type of prevalence estimate, key determinants, summary estimates of anxiety and depression prevalence, and comments.

### Quality assessment

Two reviewers (LN, PM) independently used the appraisal tool for cross-sectional studies (AXIS) to assess the quality of studies included in the review [17]. This tool was chosen because we anticipated to identify mainly cross-sectional studies. A third reviewer (EU) assessed 10% of the studies for quality assurance and was available to discuss disagreements.

We computed a summary score weighing all 20 items equally for use in the meta-regression analysis, with a higher score indicating better quality.

### Statistical analysis

Despite anticipated heterogeneity in the results of included studies, we judged that a meta-analysis that explores explanatory factors of heterogeneity is more useful than a systematic review without a quantitative synthesis of results. Ioannidis and colleagues have previously discussed these considerations in detail [18]. They argued that, providing limitations of synthesising heterogeneous results are adequately acknowledged, quantitative syntheses can provide more information than qualitative interpretation of the results, and allow for an investigation of diversity in results.

One reviewer (EU) performed random-effects meta-analyses in Stata 15 (StataCorp, College Station TX, USA) under supervision of a statistician (NM), using the *metaprop* command for meta-analysis of proportional data. We created forest plots to calculate pooled estimates where appropriate, by type of NCD.

To estimate heterogeneity, we calculated the I^2^ statistic. High heterogeneity is indicated if the I^2^ statistic is over 75% and low heterogeneity indicated if the I^2^ statistic is below 40% [19]. As we anticipated considerable heterogeneity in the prevalence estimates of depression and anxiety, we employed random-effects meta-regression analyses with the *metareg* command in Stata 15 to explore factors associated with variations in prevalence estimates of CMD. Depending on the availability of data, covariates included year of publication (continuous variable), setting (hospital vs other), country, CMD diagnostic tool (clinical assessment vs self- or interviewer-completed), and study quality (total score AXIS tool).

We investigated publication bias using funnel plots. Where the number of studies allowed for it, we performed sensitivity analyses of high-quality studies based on AXIS scores. For this purpose, we categorised studies with a quality score of 15 or higher (out of 20) as high overall quality.

## RESULTS

### Study characteristics

**Figure 1:**
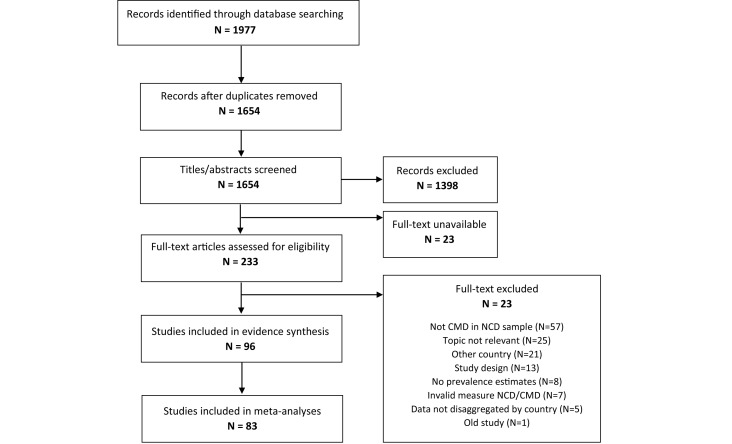
Study selection process. CMD – common mental disorders, NCD – non-communicable disease.

Out of 1654 non-duplicate records identified, 96 were included in the review and 83 were included for meta-analyses (**Figure 1**). Appendix S2 in **Online Supplementary Document** contains a full reference list of all included studies.

Table 1 describes characteristics of the included studies. Almost all studies were conducted in a hospital setting (94%). Studies were predominantly conducted in India (63%), and most studies included patients with diabetes (42%), cardiovascular disease (28%), or cancer (24%). Depression was studied more commonly than anxiety (in 99% and 35% of studies, respectively), and the HADS and PHQ-9 screening tools were most often used to screen for depression. Thirteen studies could not be included in the meta-analyses, most commonly because the authors provided only a combined estimate for depression and anxiety rather than separate estimates.

**Table 1:** Description of study characteristics

Study characteristic	No. studies (N = 96)
**Countries:**	
India	60
Pakistan	30
Bangladesh	5
All three countries	1
**Study design:**	
Cross-sectional	86
Repeated measures *(incl. before and after)*	10
**Sample size (of NCD patients):**	
20-49	9
50-200	56
>200	31
**Recruitment setting:**	
Community	6
Hospital*	90
**Common mental disorder (CMD):**	
Depression	62
Anxiety	1
Both depression and anxiety	33
**CMD screening tool:**	
Patient Health Questionnaire (PHQ-9)	20
Hospital Anxiety and Depression Scale (HADS)	14
Diagnostic and Statistical Manual of Mental Disorders (DSM)	9
Beck’s Depression Inventory (BDI)	8
Hamilton Depression/Anxiety Rating Scale (HAM)	7
Montgomery-Åsberg Depression Rating Scale (MADRS)	4
Aga Khan University Anxiety and Depression Scale (AKU-ADS)	3
Geriatric Depression Scale (GDS)	3
Siddiqui-Shah Depression Scale (SSDS)	3
Other†	10
Multiple tools	15
**Non-communicable disease:**	
Diabetes	40
Cardiovascular disease	27
Cancer	23
Respiratory conditions	4
Multiple conditions	2

*Includes patients recruited by phone or visit based on hospital records.

†Other tools: International Classification of Diseases- 10 (2), Center for Epidemiological Studies Depression Scale (2), Major Depression Inventory (1), Mini-International Neuropsychiatric Interview (1), Depression Anxiety Stress Scales (1), Hopkins Symptoms Checklist-25 (1), Brief Edinburgh Depression Scale (1), and Mini-Mental State Examination (1).

We had initially planned to summarise evidence not only for the prevalence but also the determinants of CMDs in adults with NCDs, but the literature identified was too inconsistent to provide a comprehensive overview.

### Quality assessment

**Figure 2:**
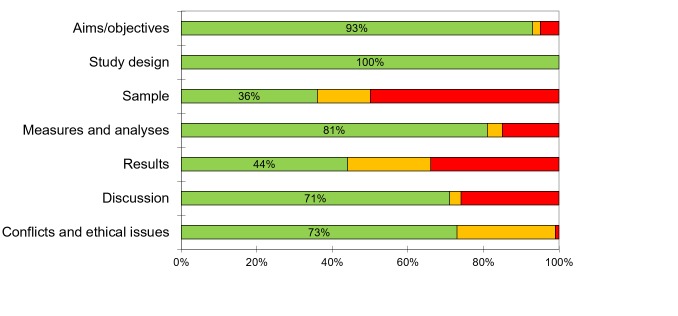
Summary of quality assessment. Green: high quality. Orange: unclear quality. Red: low quality.

The results of the quality assessment, including all 96 studies, are shown in summary in **Figure 2**, and in full in Figure S1 in **Online Supplementary Document**. The most commonly identified quality issues were the sample frame and selection process, the description of the methods and basic data, and the description of non-responders.

The stated aims and objectives for many studies referred to plans to establish the prevalence of depression and/or anxiety in the population, with the population either undefined or defined in terms of geographical setting (mostly a country or region). However, the sample strategies described were often not adequate for achieving this goal. Although almost all studies were hospital-based, study aims often implied the prevalence rates found in the studies would be relevant to the wider community. The potential and plausible differences in mental health between people attending a hospital for treatment of an NCD and those not seen in hospital were rarely considered.

In addition, people with previously diagnosed anxiety or depression, or those receiving antidepressants, were excluded from many studies that aimed to assess the prevalence of anxiety or depression in NCD populations.

Most studies did not mention how many people were missing from the source population due to non-response, and almost none of the studies provided characteristics of these people. Very few authors reported they had calculated the required sample size ahead of conducting the study. Reporting of potential conflicts of interest and confirmation of obtained ethical consent were absent or unclear in 27% of the studies.

### Meta-analysis

Eighty-three studies were included in the meta-analyses, providing 94 estimates of depression or anxiety. Some studies provided multiple estimates because they were conducted in various countries, because they reported on depression as well as anxiety, or because they included various NCD patient groups. Of the estimates for depression, six were from Bangladesh, 24 were from Pakistan and 64 from India. Of the estimates for anxiety, there were no estimates from Bangladesh, five from Pakistan, and 23 from India.

The pooled prevalence of depression from 94 estimates is 41% (95% CI = 37 to 44, I^2^ = 97%). Twenty-eight studies provided data on the prevalence of anxiety, with a pooled estimate of 29% (95% CI = 22 to 36, I^2^ = 96%).

### Patients with diabetes

**Figure 3:**
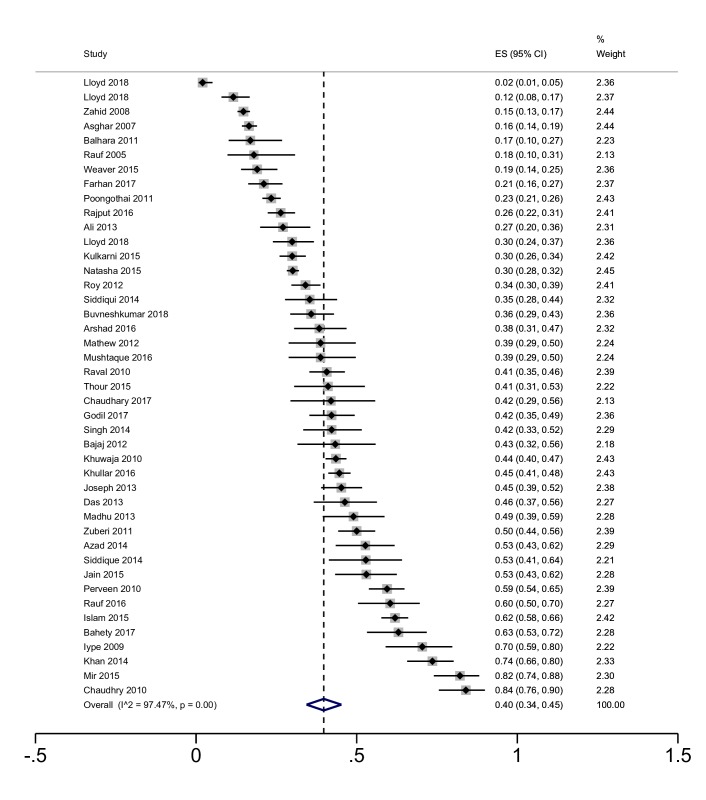
Meta-analysis of the prevalence of depression in patients with diabetes. CMD – common mental disorders, NCD – non-communicable disease. *Three estimates are included for Lloyd et al 2018 for data from Bangladesh, India, and Pakistan.

The pooled prevalence of depression from 43 estimates in 41 studies of patients with diabetes is 40% (95% CI = 34 to 45) (**Figure 3**).

Heterogeneity was extremely high (I^2^ = 97%), with prevalence rates ranging between 2% and 84%. For the smaller group of studies not from India, estimates ranged between 16% and 62% for patients with diabetes and depression in Bangladesh, and between 15% and 74% in Pakistan.

The pooled prevalence of anxiety in eight studies of patients with diabetes is 29% (95% CI = 16 to 44, I^2^ = 97%). All but two estimates were from India; two studies from Pakistan reported estimates of 12% and 58% for anxiety in patients with diabetes [20,21].

### Patients with cancer or cancer survivors

**Figure 4:**
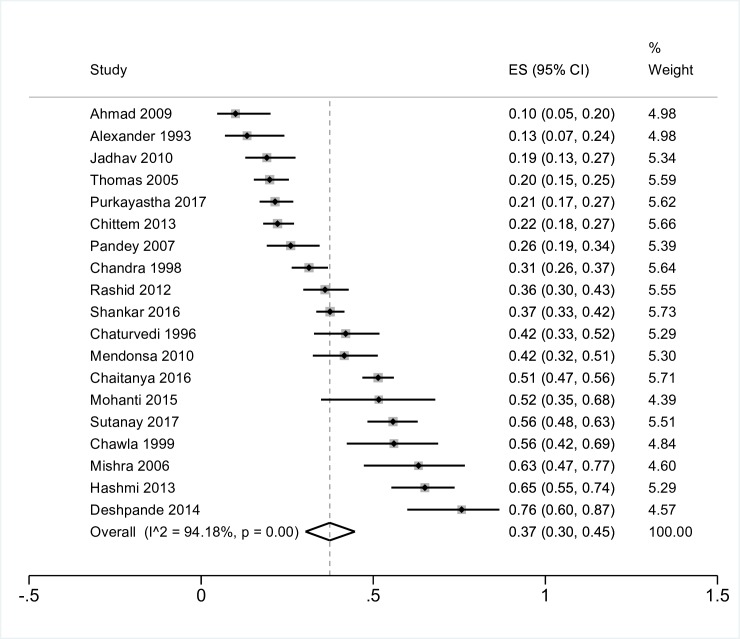
Meta-analysis of the prevalence of depression in patients with cancer CMD – common mental disorders, NCD – non-communicable disease.

Nineteen studies reported prevalence rates of patients with cancer or cancer survivors. Many studies included patients seen at inpatient or outpatient units with all types of cancer, while three studies focussed on a specific type of cancer: head and neck cancer, prostate- and breast cancer [22-24]. The pooled prevalence of depression in these studies of patients with cancer was 37% (95% CI = 30 to 45, I^2^ = 94%) (**Figure 4**). All but four estimates were from India; four studies from Pakistan reported depression prevalence rates between 10% and 65% for people with cancer.

Eleven of the studies that reported on prevalence of depression also reported on the prevalence of anxiety. Pooling the studies resulted in a combined estimate of 27% (95% CI = 16 to 40, I^2^ = 97%). Two studies were from Pakistan rather than India; these reported prevalence rates of 33% and 23% [25,26].

### Patients with cardiovascular disease

The 24 studies of CMD in patients with cardiovascular disease included studies of patients with angina (n = 1), unspecified cardiovascular or cardiac disease (n = 5), heart failure (n = 2), hypertension (n = 4), stroke (n = 6), coronary artery disease (n = 5), and myocardial infarction (n = 1). We performed meta-analyses for hypertension and stroke, as these were considered the two most homogenous chronic illness groups. Both groups consisted of patients with the same diagnosis, contrary to the other groups comprising more than one study, in which the type of diagnosis and assessment varied The pooled prevalence of depression in patients with hypertension was 38% (95% CI = 32 to 45, I^2^ = 91%) and the pooled prevalence of depression in patients with a previous stroke was 39% (95% CI = 23 to 56, I^2^ = 96%).

The reported prevalence of anxiety, which was estimated in patients with stroke, coronary artery disease, myocardial infarction, and unspecified heart disease, ranged from 3% to 58%.

### Patients with chronic respiratory conditions

**Figure 5:**
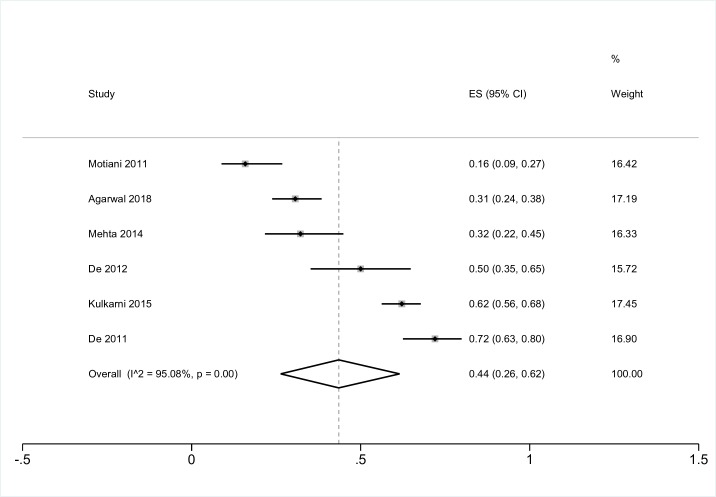
Meta-analysis of the prevalence of depression in patients with COPD CMD – common mental disorders, NCD – non-communicable disease, COPD – chronic obstructive pulmonary disease.

Seven studies, six from India and one from Pakistan, reported on the prevalence of CMD in patients with chronic respiratory conditions. One of these studies measured a prevalence of depression of 30% in patients with bronchial asthma [27], one reported 19% anxiety among 59 patients with COPD [28], and six reported on the prevalence of depression in patients with COPD (**Figure 5**) (pooled estimate 44%, 95% CI = 26 to 62, I^2^ 95%). The study of depression in patients with COPD from Pakistan reported a prevalence of 15% [29].

### Risk of publication bias

To assess the risk of bias we constructed and visually inspected a funnel plot of prevalence estimates of depression, to which all but one of the studies contributed data (Figure S2 in **Online Supplementary Document**). Visual inspection of the funnel plot shows a flat funnel-shaped distribution due to the large amount of relatively small studies, the lack of medium-sized samples, and a few very large studies. The studies with large sample sizes tend to have a lower prevalence rate of depression.

### Sensitivity analysis of high-quality studies

Including 29 high quality studies only (quality score ≥15 out of 20), the pooled prevalence of depression for all NCD categories is 35% (95% CI = 0.30 to 0.41, I^2^ = 98%). Only three studies reporting on the prevalence of anxiety in diabetes, cancer, and stroke, respectively, were rated high quality, producing a pooled estimate of 33% (95% CI = 0.10 to 0.62, I^2^ = 99%).

### Meta-regression

Restricted by the availability of reliable data on patient and study characteristics, we included the following variables in the meta-regression analysis for depression prevalence: year of publication, country, NCD category, sample size (categorical), type of CMD diagnostic tool (diagnostic interview vs screening tool), and the total score of study quality.

The apparent associations between a higher prevalence of depression and a more recent publication date and lower quality score had limited impact on explaining heterogeneity (adjusted r^2^ = 0.4%). None of the covariates were statistically significant (Table S1 in **Online Supplementary Document**).

For the meta-regression analysis modelling anxiety prevalence, year of publication, sample size, and quality score were included. For all other variables, some categories had cell counts of less than five and were therefore not included. These covariates explained more of the heterogeneity for anxiety prevalence (adjusted r^2^ = 11%) than for depression. However, again none of the covariates were statistically significant predictors of prevalence estimates (Table S2 in **Online Supplementary Document**).

## DISCUSSION

### Summary of findings

Ninety-six studies reported on the prevalence of CMDs in patients with NCDs in Bangladesh, India, or Pakistan, in predominantly hospital-based studies. The pooled estimate of depression was 40% in patients with diabetes, 37% in patients with cancer, 38% in patients with hypertension, 39% in patients with stroke, and 44% in patients with COPD. The pooled prevalence of anxiety was 29% in patients with diabetes and 27% in patients with cancer.

For all four NCDs, findings were extremely heterogeneous, for example, the prevalence of depression in patients with diabetes ranged from 2% to 84%. The meta-regression analyses did not reveal study characteristics associated with the reported prevalence estimates of depression and anxiety. Study country was also not associated with prevalence of either depression or anxiety. However, a sensitivity analysis with high quality studies only estimated a pooled prevalence of depression across NCDs of 35%, lower than the 41% found in the main analysis.

### Limitations

For studies of prevalence, which summarise data from observational studies of patients with varying characteristics and from a wide range of settings, heterogeneity in the estimates is to be expected. However, despite our efforts to conduct a high-quality systematic review, it is likely that the quality and heterogeneity of the included studies affect the reliability of the findings.

Limitations of the primary evidence included a mismatch between intended study population and sampling strategies, no mention of participants who did not complete questionnaires or people who were approached but did not join the study, and limited descriptions of study methods and participant characteristics.

Most studies excluded patients who had a diagnosis of depression or anxiety, or who were already receiving medication to treat a CMD prior to inclusion in the study. Estimates for these studies therefore reflect undiagnosed depression or anxiety, rather than the point prevalence estimate of common mental disorder in the total population of patients with NCDs. Prevalence rates we reported are therefore likely to be underestimates of the true prevalence.

Although we explored variation in estimates related to whether a diagnostic interview or screening tool was used to identify depression and/ or anxiety, the type of screening tool, translation and validation of the tool, and how and by whom screening was performed, may explain some of the heterogeneity identified. Many different screening tools were identified (**Table 1**). The most commonly used tools, such as the PHQ-9, HADS, MADRS, BDI, and HAM have been widely translated and validated. However, not all studies reported whether an appropriately translated version was used. Some studies used less established measures without reporting on validation or translation of the tool, and some studies used multiple tools. Authors generally conducted studies in large, metropolitan hospitals with the resources to conduct research. Only 6 were community studies. This limits the generalisability of our findings, as the group of patients with NCDs attending these settings are likely to differ from those who attend smaller, regional hospitals or those who do not regularly visit the hospital due to either limited access to health care or a lower severity of illness. However, prevalence rates were still found to be high in community studies included in our review. Depression in patients with diabetes for example was found to be between 15% and 36%. A review of studies on diabetes and depression internationally reported a prevalence of 20% in 12 community studies, compared to 32% in studies with a clinic setting [30].

It is possible that we missed relevant studies, particularly those published in journals specific to South-Asia. Although we searched in databases from Bangladesh, India, and Pakistan, these databases are more difficult to search due to their limited search interface. Our decision not to include grey literature is also likely to have reduced the number of studies we identified.

Methodology for systematic reviews and meta-analyses of prevalence studies is underdeveloped compared to methods and guidance for evidence synthesis of randomised controlled trials and treatment studies. To our knowledge, no widely accepted and validated tool for quality assessment of prevalence studies is available. Although we used a quality assessment tool developed for use with cross-sectional studies,[17] we found that the nature of the included studies meant assessment was subjective and extremely time-consuming. We see room for improvement of the tool, by providing more explicit guidance to the different items (for example, when are ‘basic data adequately described’?), and by re-assessing the weight given to certain indicators of study quality (for example, three out of 20 items address response rate and non-responder bias). We used summary scores of study quality weighing all items equally, as the tool developers provided no guidance for calculating summary scores.

Some studies provided multiple estimates, either for multiple NCDs or for anxiety as well as depression. By including these estimates in one meta-regression analysis, we will have underestimated the between-study variation. Similarly, the pooled estimate for depression in diabetes includes three estimates from the same study in three different countries. As it is highly unlikely that this would have a substantial impact on the results, we have not adjusted for this.

### Implications for research and practice

Given the heterogeneity of our findings and the quality issues identified in many of the included studies, the pooled estimates should not be interpreted as true estimates of the average prevalence rate of CMDs in people with NCDs in these three South Asian countries. Most of the estimates provided reflect the burden of disease in a hospital population with no previous diagnosis or treatment of mental ill health. The meta-regression analyses explained little of the variance in prevalence rates identified. Our findings also do not explain how prevalence rates of CMDs vary between patient groups. For example, most studies we included had a mixed sample of men and women and estimates of depression and anxiety are likely to differ by gender.

Our systematic review shows that gaps remain in knowledge on the burden of disease for CMDs and NCDs in Bangladesh, India, and Pakistan. Large and well-conducted community surveys, representative of the entire population of patients with NCDs, would make a valuable contribution to research on the burden of physical and mental comorbidities in South Asia, and provide robust data to policymakers to map demand for services. Worldwide case-control studies which have aimed to investigate associations between psychosocial factors and heart disease include the INTERHEART and INTERSTROKE studies [31,32]. For South Asia, the INTERHEART study reported a 30% prevalence rate of depression for those who suffered from a myocardial infarction, compared to 19% depression in the control group, around the year 2000 [30]. In the INTERSTROKE study, patients from South Asia with acute stroke were found to be at higher odds of psychosocial stress, including depression, than a control group [32].

CMDs and NCDs have a large impact on people’s quality of life and living with anxiety or depression on top of a long-term physical illness can lead to greater physical deterioration, functional impairment, lower adherence to medication, and premature mortality [33,34]. However, successful implementation of integrated care for people with NCDs, including CMDs, faces many barriers. A recent review pointed towards a lack of commitment from actors at all levels, which translates into limited human resources and skills, insufficient coordination of policies, and low workforce preparedness [35]. If mental and physical comorbidity is as common as suggested by our findings, practitioners and policymakers must act at the clinical and the policy level, to make high quality and tailored mental health care available to patients with NCDs. This is particularly relevant in low- and middle-income countries, where current resources and infrastructure may not be able to accommodate such comprehensive mental health care.

## CONCLUSIONS

Although the wide variation in estimates of CMDs limits our ability to draw definite conclusions on prevalence rates of CMDs in these patient groups, it is clear that the prevalence of depression and anxiety is high in people with NCDs in South Asia. Prevalence rates between 20 and 30% are among the lower-end estimates and constitute a great burden of mental ill health for which there is currently little attention in either research, practice, or policy.

## Additional material

Online Supplementary Document
