# Supplementary material for: A systematic review and meta-analysis of the prevalence of common mental disorders in people with non-communicable diseases in Bangladesh, India, and Pakistan
Source: J Glob Health. 2019 Dec 16;9(2):020417. doi: 10.7189/jogh.09.020417 (PMC6925965; doi:10.7189/jogh.09.020417)
Supplement: Online Supplementary Document [file jogh-09-020417-s001.pdf]

Online Supplementary Documents

Appendix S1. Search strategies

Date of completion: 04-10-18

Databases:

- BRAC Research & Publication http://research.brac.net/new/publications (accessed 02-10-2018)
- Cochrane Database of Systematic Reviews : Issue 7 of 12, July 2018
- Database of Abstracts of Reviews of Effect (Wiley): Issue 2 of 4, April 2015
- Global Health 1910 to 2018 Week 25
- Global Index Medicus (World Health Organization) Libraries (Inception to 03-10-2018)
- Health Technology Assessment Database (Wiley): Issue 4 of 4, October 2016
- IndMED (ICMR-NIC) 1985 – 02-10-2018
- Ovid MEDLINE(R) <1946 to June Week 4 2018>
- Ovid MEDLINE(R) In-Process & Other Non-Indexed Citations <July 02, 2018>
- Ovid MEDLINE(R) Epub Ahead of Print <July 02, 2018>
- PakMediNet (PakCyber) Inception – 13-09-2018
- PsycINFO 1806 to June Week 4 2018
- World Bank Group Research and Publications: Documents and Reports http://documents.worldbank.org/curated/en/docadvancesearch/ (accessed 13-09-2018)

BRAC Research & Publication http://research.brac.net/new/publications (accessed 02-10-2018)
[note, search terms entered individually into the web page, one-by-one]

cancer OR neoplasm OR diabetes OR pulmonary OR lung OR respiratory OR bronchitis OR Emphysema OR stroke OR cardiovascular OR vascular OR cardiac OR coronary OR arterial OR hypertension

Cochrane Database of Systematic Reviews : Issue 7 of 12, July 2018
Search Name: S Asia Common Mental Disorders Non Comm Diseases 25-06-2018
Last Saved: 25/06/2018 13:24:35.571

ID	Search
#1	MeSH descriptor: [Asia, Western] this term only
#2	MeSH descriptor: [Bangladesh] this term only
#3	MeSH descriptor: [India] explode all trees
#4	MeSH descriptor: [Pakistan] this term only
#5	MeSH descriptor: [Bhutan] this term only
#6	MeSH descriptor: [Afghanistan] this term only
#7	MeSH descriptor: [Nepal] this term only
#8	MeSH descriptor: [Indian Ocean Islands] explode all trees
#9	(Indian or Indians) not ("west indian*" or "american indian*"):ti,ab,kw
#10	("south asia*" or india or bangladesh* or pakistan*):ti,ab,kw
#11	(Bhutan* or afghanistan* or maldive* or nepal* or "sri lanka*"):ti,ab,kw
#12	{or #1-#11}
#13	MeSH descriptor: [Comorbidity] explode all trees
#14	(co-morbid* or comorbid* or multi-morbid* or multimorbid*):ti,ab,kw
#15	MeSH descriptor: [Chronic Disease] this term only
#16	(("long term" or chronic) near/3 (disease* or illness* or condition* or disorder*)):ti,ab,kw
#17	MeSH descriptor: [Diabetes Mellitus] explode all trees
#18	MeSH descriptor: [Cardiovascular Diseases] explode all trees
#19	MeSH descriptor: [Pulmonary Disease, Chronic Obstructive] explode all trees
#20	MeSH descriptor: [Stroke] 2 tree(s) exploded
#21	MeSH descriptor: [Neoplasms] explode all trees
#22	diabet*:ti,ab,kw
#23	(Aneurysm* or Atherosclerosis or Cardio* or endocard* or pericard* or heart or cardiac or valvular or coronary or arterial or vascular or hypertension):ti,ab,kw
#24	((long-term or chronic*) near/2 (airway* or pulmonar* or airflow* or lung*) near/2 Obstruct*):ti,ab,kw

#25 (pulmonar* near/2 emphysem*):ti,ab,kw
#26 ((long-term or chronic*) near/5 (bronchitis or respirat*)):ti,ab,kw
#27 ((long-term or Chronic*) near/2 lung* near/5 (condition* or disease* or symptom* or problem* or failure*)):ti,ab,kw
#28 (respiratory near/2 (condition* or disease* or symptom* or problem*)):ti,ab,kw
#29 (stroke or poststroke or post-stroke or cerebrovasc* or brain vasc* or cerebral vasc* or apoplex*):ti,ab,kw
#30 ((brain* or cerebr* or cerebell* or intracran* or intracerebral) near/2 (isch*emi* or infarct* or thrombo* or emboli* or occlus*)):ti,ab,kw
#31 ((brain* or cerebr* or cerebell* or intracerebral or intracranial or subarachnoid) near/2 (h*emorrhage* or h*ematoma* or bleed*)):ti,ab,kw
#32 (transi* near/3 isch*em* near/3 attack*):ti,ab,kw
#33 (cancer* or carcinoma* or tumor* or tumour* or neoplasm* or malignan*):ti,ab,kw
#34 {or #13-#33}
#35 (somat* or hysteri* or briquet or multisomat* or MUPs or "medically unexplained" or depress* or MDD or anxi* or phobia or phobic):ti,ab,kw
#36 ("stress syndrome*" or "distress syndrome*" or "pain disorder*" or dissociation* or dissociative*):ti,ab,kw
#37 ((affective or stress or cognitive or cognition or personality or impulse or mood or paranoid or psychotic or neurologic* or nervous or eating) near/1 (disorder* or illness* or disease*)):ti,ab,kw
#38 ((bipolar or behavio*ral or obsessive or compulsive or agoraphobi* or delusional) near/1 (disorder* or illness* or disease*)):ti,ab,kw
#39 (OCD or "obsess* compulsi*" or GAD or stress reaction* or acute stress or neuros*s or neurotic):ti,ab,kw
#40 (stress near/5 (chronic* or long-term or severe or mental* or psyc*)):ab
#41 stress:ti
#42 MeSH descriptor: [Anxiety Disorders] explode all trees
#43 MeSH descriptor: [Depression] this term only
#44 MeSH descriptor: [Depressive Disorder] this term only
#45 MeSH descriptor: [Depression, Postpartum] this term only
#46 MeSH descriptor: [Depressive Disorder, Treatment-Resistant] this term only
#47 MeSH descriptor: [Stress, Psychological] explode all trees
#48 {or #35-#47}
#49 #12 and #34 and #48

Database of Abstracts of Reviews of Effect (Wiley): Issue 2 of 4, April 2015
Same strategy as Cochrane Database of Systematic Reviews : Issue 7 of 12, July 2018

Global Health 1910 to 2018 Week 25
Database: Global Health <1910 to 2018 Week 25>

1 ((Indian or Indians) not ("west indian*" or "american indian*")).ti,ab,in. (42039)
2 india.ti,ab,in. (188992)
3 ("south asia*" or bangladesh* or pakistan*).ti,ab,in. (36364)
4 (Bhutan* or afghanistan* or maldive* or nepal* or "sri lanka*").ti,ab,in. (14098)
5 exp south asia/ (144606)
6 or/1-5 [All S Asia World Bank] (256984)
7 (co-morbid* or comorbid*).ti,ab. (18954)
8 (multi-morbid* or multimorbid*).ti,ab. (520)
9 (("long term" or chronic) adj3 (disease? or illness* or condition? or disorder?)).tw. (59861)
10 diabet*.tw. (118256)
11 (Aneurysm* or Atherosclerosis or Cardio* or endocard* or pericard*).tw. (123560)
12 (heart or cardiac or valvular or coronary or arterial or vascular or hypertension).tw. (188948)
13 ((long-term or chronic*) adj2 (airway* or pulmonar* or airflow* or lung*) adj2 Obstruct*).tw. (6955)
14 (pulmonar* adj2 emphysem*).tw. (1701)
15 ((long-term or chronic*) adj5 (bronchitis or respirat*)).tw. (5131)
16 ((long-term or Chronic*) adj2 lung* adj5 (condition* or disease* or symptom* or problem* or failure*)).tw. (1685)
17 (respiratory adj2 (condition* or disease* or symptom* or problem*)).tw. (66802)

18 (stroke or poststroke or post-stroke or cerebrovasc* or brain vasc* or cerebral vasc* or apoplex*).tw. (20901)
19 ((brain? or cerebr* or cerebell* or intracran* or intracerebral) adj2 (isch?emi* or infarct* or thrombo* or emboli* or occlus*)).tw. (4648)
20 ((brain? or cerebr* or cerebell* or intracerebral or intracranial or subarachnoid) adj2 (h?emorrhage? or h?ematoma* or bleed*)).tw. (2665)
21 (transi* adj3 isch?em* adj3 attack?).tw. (564)
22 (cancer* or carcinoma? or tumor? or tumour? or neoplasm? or malignan*).tw. (278594)
23 or/7-22 [Chronic or Multimorbid incl Cancer] (671851)
24 (somatoform or somatiz* or somatis* or hysteri* or briquet or multisomat* or multi somat* or MUPs or medically unexplained).tw. (1250)
25 ((dissociative adj3 (disorder* or reaction*)) or dissociation).tw. (4069)
26 (affective* adj (disorder? or disease? or illness* or symptom?)).tw. (1685)
27 ((stress or cognitive or cognition or personality or impulse control or mood or paranoid or psychotic or neurologic* or nervous or eating) adj (disorder? or illness* or disease?)).tw. (25226)
28 ((bipolar or behavio?ral or obsessive or compulsive or panic or mood or agoraphobi* or delusional) adj (disorder? or illness* or disease?)).tw. (3798)
29 (OCD or obsess*-compulsi* or GAD or stress reaction? or acute stress or neuros#s or neurotic).tw. (3144)
30 (stress syndrome? or distress syndrome? or pain disorder?).tw. (2738)
31 (depressi* or depressed or MDD or anxi* or phobia or phobic).tw. (54609)
32 (stress adj5 (chronic* or long-term or severe or mental* or psyc*)).ab. (5983)
33 stress.ti. (22165)
34 or/24-33 [CMDs] (105390)
35 6 and 23 and 34 [S Asia NCDs CMDs] (2020)

36 (incidence* adj5 (somatoform or somatiz* or somatis* or hysteri* or briquet or multisomat* or multi somat* or MUPs or medically unexplained or ((dissociative adj3 (disorder* or reaction*)) or dissociation) or (affective* adj (disorder? or disease? or illness* or symptom?)) or ((stress or cognitive or cognition or personality or impulse control or paranoid or psychotic or neurologic* or nervous or eating) adj (disorder? or illness* or disease?)) or ((bipolar or behavio?ral or obsessive or compulsive or panic or mood or agoraphobi* or phobic or delusional) adj (disorder? or illness* or disease?)) or (OCD or obsess*-compulsi* or GAD or stress reaction? or acute stress or neuros#s or neurotic) or (stress syndrome? or distress syndrome? or pain disorder?) or (depressi* or depressed or MDD or anxi* or phobia or phobic))).tw,kw. (846)

37 (prevalen* adj5 (somatoform or somatiz* or somatis* or hysteri* or briquet or multisomat* or multi somat* or MUPs or medically unexplained or ((dissociative adj3 (disorder* or reaction*)) or dissociation) or (affective* adj (disorder? or disease? or illness* or symptom?)) or ((stress or cognitive or cognition or personality or impulse control or paranoid or psychotic or neurologic* or nervous or eating) adj (disorder? or illness* or disease?)) or ((bipolar or behavio?ral or obsessive or compulsive or panic or mood or agoraphobi* or phobic or delusional) adj (disorder? or illness* or disease?)) or (OCD or obsess*-compulsi* or GAD or stress reaction? or acute stress or neuros#s or neurotic) or (stress syndrome? or distress syndrome? or pain disorder?) or (depressi* or depressed or MDD or anxi* or phobia or phobic))).tw,kw. (3365)

38 (determinant* adj5 (somatoform or somatiz* or somatis* or hysteri* or briquet or multisomat* or multi somat* or MUPs or medically unexplained or ((dissociative adj3 (disorder* or reaction*)) or dissociation) or (affective* adj (disorder? or disease? or illness* or symptom?)) or ((stress or cognitive or cognition or personality or impulse control or paranoid or psychotic or neurologic* or nervous or eating) adj (disorder? or illness* or disease?)) or ((bipolar or behavio?ral or obsessive or compulsive or panic or mood or agoraphobi* or phobic or delusional) adj (disorder? or illness* or disease?)) or (OCD or obsess*-compulsi* or GAD or stress reaction? or acute stress or neuros#s or neurotic) or (stress syndrome? or distress syndrome? or pain disorder?) or (depressi* or depressed or MDD or anxi* or phobia or phobic))).tw,kw. (163)

39 (cohort* adj5 (somatoform or somatiz* or somatis* or hysteri* or briquet or multisomat* or multi somat* or MUPs or medically unexplained or ((dissociative adj3 (disorder* or reaction*)) or dissociation) or (affective* adj (disorder? or disease? or illness* or symptom?)) or ((stress or cognitive or cognition or personality or impulse control or paranoid or psychotic or neurologic* or nervous or eating) adj (disorder? or illness* or disease?)) or ((bipolar or behavio?ral or obsessive or compulsive or panic or mood or agoraphobi* or phobic or delusional) adj (disorder? or illness* or disease?)) or (OCD or obsess*-compulsi* or GAD or stress reaction? or acute stress or neuros#s or neurotic) or (stress syndrome? or distress syndrome? or pain disorder?) or (depressi* or depressed or MDD or anxi* or phobia or phobic))).tw,kw. (488)

40 (observational* adj5 (somatoform or somatiz* or somatis* or hysteri* or briquet or multisomat* or multi somat* or MUPs or medically unexplained or ((dissociative adj3 (disorder* or reaction*)) or dissociation) or (affective* adj (disorder? or disease? or illness* or symptom?)) or ((stress or cognitive or cognition or personality or impulse control or paranoid or psychotic or neurologic* or nervous or eating) adj (disorder? or illness* or disease?)) or ((bipolar or behavio?ral or obsessive or compulsive or panic or mood or agoraphobi* or phobic or delusional) adj (disorder? or illness* or disease?)) or (OCD or obsess*-compulsi* or GAD or stress reaction? or acute stress or neuros#s or neurotic) or (stress syndrome? or distress syndrome? or pain disorder?) or (depressi* or depressed or MDD or anxi* or phobia or phobic))).tw,kw. (44)

41 ("risk factor*" adj5 (somatoform or somatiz* or somatis* or hysteri* or briquet or multisomat* or multi somat* or MUPs or medically unexplained or ((dissociative adj3 (disorder* or reaction*)) or dissociation) or (affective* adj (disorder? or disease? or illness* or symptom?)) or ((stress or cognitive or cognition or personality or impulse control or paranoid or psychotic or neurologic* or nervous or eating) adj (disorder? or illness* or disease?)) or ((bipolar or behavio?ral or obsessive or compulsive or panic or mood or agoraphobi* or phobic or delusional) adj (disorder? or illness* or disease?)) or (OCD or obsess*-compulsi* or GAD or stress reaction? or acute stress or neuros#s or neurotic) or (stress syndrome? or distress syndrome? or pain disorder?) or (depressi* or depressed or MDD or anxi* or phobia or phobic))).tw,kw. (1892)

42 (epidemiolog* adj5 (somatoform or somatiz* or somatis* or hysteri* or briquet or multisomat* or multi somat* or MUPs or medically unexplained or ((dissociative adj3 (disorder* or reaction*)) or dissociation) or (affective* adj (disorder? or disease? or illness* or symptom?)) or ((stress or cognitive or cognition or personality or impulse control or paranoid or psychotic or neurologic* or nervous or eating) adj (disorder? or illness* or disease?)) or ((bipolar or behavio?ral or obsessive or compulsive or panic or mood or agoraphobi* or phobic or delusional) adj (disorder? or illness* or disease?)) or (OCD or obsess*-compulsi* or GAD or stress reaction? or acute stress or neuros#s or neurotic) or (stress syndrome? or distress syndrome? or pain disorder?) or (depressi* or depressed or MDD or anxi* or phobia or phobic))).tw. (1853)

43 ((longitudinal or prospective or retrospective) adj5 (somatoform or somatiz* or somatis* or hysteri* or briquet or multisomat* or multi somat* or MUPs or medically unexplained or ((dissociative adj3 (disorder* or reaction*)) or dissociation) or (affective* adj (disorder? or disease? or illness* or symptom?)) or ((stress or cognitive or cognition or personality or impulse control or paranoid or psychotic or neurologic* or nervous or eating) adj (disorder? or illness* or disease?)) or ((bipolar or behavio?ral or obsessive or compulsive or panic or mood or agoraphobi* or phobic or delusional) adj (disorder? or illness* or disease?)) or (OCD or obsess*-compulsi* or GAD or stress reaction? or acute stress or neuros#s or neurotic) or (stress syndrome? or distress syndrome? or pain disorder?) or (depressi* or depressed or MDD or anxi* or phobia or phobic))).tw. (683)

44 (case-control* adj5 (somatoform or somatiz* or somatis* or hysteri* or briquet or multisomat* or multi somat* or MUPs or medically unexplained or ((dissociative adj3 (disorder* or reaction*)) or dissociation) or (affective* adj (disorder? or disease? or illness* or symptom?)) or ((stress or cognitive or cognition or personality or impulse control or paranoid or psychotic or neurologic* or nervous or eating) adj (disorder? or illness* or disease?)) or ((bipolar or behavio?ral or obsessive or compulsive or panic or mood or agoraphobi* or phobic or delusional) adj (disorder? or illness* or disease?)) or (OCD or obsess*-compulsi* or GAD or stress reaction? or acute stress or neuros#s or neurotic) or (stress syndrome? or distress syndrome? or pain disorder?) or (depressi* or depressed or MDD or anxi* or phobia or phobic))).tw. (173)

45 ((follow-up or follow-on) adj5 (somatoform or somatiz* or somatis* or hysteri* or briquet or multisomat* or multi somat* or MUPs or medically unexplained or ((dissociative adj3 (disorder* or reaction*)) or dissociation) or (affective* adj (disorder? or disease? or illness* or symptom?)) or ((stress or cognitive or cognition or personality or impulse control or paranoid or psychotic or neurologic* or nervous or eating) adj (disorder? or illness* or disease?)) or ((bipolar or behavio?ral or obsessive or compulsive or panic or mood or agoraphobi* or phobic or delusional) adj (disorder? or illness* or disease?)) or (OCD or obsess*-compulsi* or GAD or stress reaction? or acute stress or neuros#s or neurotic) or (stress syndrome? or distress syndrome? or pain disorder?) or (depressi* or depressed or MDD or anxi* or phobia or phobic))).tw. (539)

46 (cross-sectional* adj5 (somatoform or somatiz* or somatis* or hysteri* or briquet or multisomat* or multi somat* or MUPs or medically unexplained or ((dissociative adj3 (disorder* or reaction*)) or dissociation) or (affective* adj (disorder? or disease? or illness* or symptom?)) or ((stress or cognitive or cognition or personality or impulse control or paranoid or psychotic or neurologic* or nervous or eating) adj (disorder? or illness* or disease?)) or ((bipolar or behavio?ral or obsessive or compulsive or panic or mood or agoraphobi* or phobic or delusional) adj (disorder? or illness* or disease?)) or (OCD or obsess*-compulsi* or GAD or stress reaction? or acute stress or neuros#s or neurotic) or (stress syndrome? or distress syndrome? or pain disorder?) or (depressi* or depressed or MDD or anxi* or phobia or phobic))).tw. (382)

47 or/36-46 [CMD Prevalence or obs studies] (8286)
48 systematic reviews/ or meta-analysis/ (30922)
49 ((systematic or evidence) adj3 review?).ti. (18397)
50 (meta-analysis or meta-synthesis).ti. (15888)
51 evidence synthes?s.ti. (50)
52 (medline or pubmed or "literature search*" or "search strateg*").ab. (27632)
53 or/48-52 [Systematic Reviews] (45178)
54 6 and 23 and 34 and 53 [S Asia NCDs CMDs and Systematic Reviews] (34)
55 6 and 23 and 47 [S Asia NCDs CMDs Prevalence or Obs studies] (105)
56 54 or 55 (134)
57 limit 56 to yr="1990 -Current" (130)
58 (rat or rats or mice or mouse).ti. (201913)
59 ((child* or infant* or baby or babies or preterm* or adolesc* or newborn* or pediatric* or paediatri* or neonate* or teen* or schoolchild*) not (adult or elderly or geriatric*)).ti. (234666)
60 57 not (58 or 59) [Final search animals and children removed] (121)

Global Index Medicus (World Health Organization) Inception to 03-10-18
Advanced Search

(tw:(cancer OR neoplasm OR diabetes OR pulmonary OR lung OR respiratory OR bronchitis OR Emphysema OR stroke OR cardiovascular OR vascular OR cardiac OR coronary OR arterial OR hypertension)) AND (tw:(depression OR depressed OR depressive OR anxiety OR stress OR distress OR phobia OR affective OR somatoform OR behavioral OR behavioural OR panic OR compulsive OR cognitive)) AND (tw:(Prevalence OR Incidence OR Epidemiology OR Observational OR cohort OR cross-sectional OR case-control)) AND (tw:(india* OR bangladesh* OR pakistan*))

Note: Search results were filtered to include all databases except Medline. The following databases within GIM contained records matching the search query:

IMSEAR (South-EastAsia) (165)

IMEMR (Eastern Mediterranean) (55)

LILACS (Americas) (18)

WPRIM (Western Pacific) (14)

AIM (Africa) (1)

Health Technology Assessment Database (Wiley): Issue 4 of 4, October 2016
Same strategy as Cochrane Database of Systematic Reviews : Issue 7 of 12, July 2018

IndMED (ICMR-NIC) 1985 - 02-10-18
Advanced Search Mode

1. cancer OR neoplasm OR diabetes OR pulmonary OR lung OR respiratory OR bronchitis OR Emphysema OR stroke OR cardiovascular OR vascular OR cardiac OR coronary OR arterial OR hypertension [searched in Anywhere field]
2. depression OR depressed OR depressive OR anxiety OR stress OR distress OR phobia OR affective OR somatoform OR behavioral OR behavioural OR panic OR compulsive OR cognitive [searched in Anywhere field]
3. Prevalence OR Incidence OR Epidemiology OR Observational OR cohort OR cross-sectional OR case-control [searched in Anywhere field]
4. 1 AND 2 AND 3

Note: this strategy represents the overall search strategy. In practice, the search was run as three separate searches with to reduce the number of terms in the first search line. This enabled searches to be executed fully without technical issues. Search terms were split between the three searches as follows: (1) Cancer OR Diabetes terms (2) COPD terms (3) CVD OR Stroke terms. Search lines 2 and 3 were the same for all searches. Search line 4 has been added to indicate the selection of 'AND's in the Advance Search form

Ovid MEDLINE(R) <1946 to June Week 4 2018>

1 ((Indian or Indians) not ("west indian*" or "american indian*")).ti,ab,in,kf. (73100)
2 india.ti,ab,in,kf. (284529)
3 ("south asia*" or bangladesh* or pakistan*).ti,ab,in,kf. (45349)
4 bangladesh/ or exp india/ or pakistan/ (113681)
5 asia, western/ or bangladesh/ or bhutan/ or exp india/ or afghanistan/ or nepal/ or pakistan/ or sri lanka/ (129398)
6 exp Indian Ocean Islands/ (10434)
7 (Bhutan* or afghanistan* or maldive* or nepal* or "sri lanka*").ti,ab,in,kf. (21618)
8 or/1-7 [All S Asia World Bank] (404711)
9 comorbidity/ or multimorbidity/ (93912)
10 (co-morbid* or comorbid*).ti,ab,kf. (118030)
11 (multi-morbid* or multimorbid*).ti,ab,kf. (2888)
12 Chronic Disease/ (248464)
13 (("long term" or chronic) adj3 (disease? or illness* or condition? or disorder?)).tw,kf. (258009)
14 exp Diabetes Mellitus/ (383482)
15 exp Cardiovascular Diseases/ (2194836)
16 exp Pulmonary Disease, Chronic Obstructive/ (48501)
17 exp Stroke/ (114253)
18 diabet*.tw,kf. (488856)
19 (Aneurysm* or Atherosclerosis or Cardio* or endocard* or pericard*).tw,kf. (831750)
20 (heart or cardiac or valvular or coronary or arterial or vascular or hypertension).tw,kf. (1939176)
21 ((long-term or chronic*) adj2 (airway* or pulmonar* or airflow* or lung*) adj2 Obstruct*).tw. (40044)
22 (pulmonar* adj2 emphysem*).tw. (4390)
23 ((long-term or chronic*) adj5 (bronchitis or respirat*)).tw,kf. (22988)
24 ((long-term or Chronic*) adj2 lung* adj5 (condition* or disease* or symptom* or problem* or failure*)).tw,kf. (11660)
25 (respiratory adj2 (condition* or disease* or symptom* or problem*)).tw,kf. (47507)
26 (stroke or poststroke or post-stroke or cerebrovasc* or brain vasc* or cerebral vasc* or apoplex*).tw. (218286)
27 ((brain? or cerebr* or cerebell* or intracran* or intracerebral) adj2 (isch?emi* or infarct* or thrombo* or emboli* or occlus*)).tw. (67945)
28 ((brain? or cerebr* or cerebell* or intracerebral or intracranial or subarachnoid) adj2 (h?emorrhage? or h?ematoma* or bleed*)).tw. (48231)
29 (transi* adj3 isch?em* adj3 attack?).tw. (11531)
30 exp neoplasm/ (3052146)
31 (cancer* or carcinoma? or tumor? or tumour? or neoplasm? or malignan*).tw,kf. (2591076)
32 or/9-31 [Chronic or Multimorbid incl Cancer] (7188621)
33 exp anxiety disorders/ (74194)
34 Depression/ (102161)
35 exp Stress, Psychological/ (115894)
36 depressive disorder/ or depression, postpartum/ or depressive disorder, treatment-resistant/ (73389)
37 or/33-36 [CMD MeSH] (326892)
38 incidence/ or prevalence/ (462052)
39 observational study/ (48677)
40 epidemiologic studies/ or case-control studies/ or cohort studies/ or follow-up studies/ or exp longitudinal studies/ or prospective studies/ or retrospective studies/ or cross-sectional studies/ (2154542)
41 exp population characteristics/ (1764233)
42 risk factors/ (726399)
43 or/38-42 [Prevalence incidence Obs Studies MeSH] (3741412)
44 37 and 43 [Prevalence or Obs studies of NCDs MeSH] (115481)
45 (prevalen* adj5 (somatoform or somatiz* or somatis* or hysteri* or briquet or multisomat* or multi somat* or MUPs or medically unexplained or ((dissociative adj3 (disorder* or reaction*)) or dissociation) or (affective* adj (disorder? or disease? or illness* or symptom?)) or ((stress or cognitive or cognition or personality or impulse control or paranoid or psychotic or neurologic* or nervous or eating) adj (disorder? or illness* or disease?)) or ((bipolar or behavio?ral or obsessive or compulsive or panic or mood or agoraphobi* or phobic or delusional) adj (disorder? or illness* or disease?)) or (OCD or obsess*-compulsi* or GAD or stress reaction? or acute stress or neuros#s or neurotic) or (stress syndrome? or distress syndrome? or pain disorder?) or (depressi* or depressed or MDD or anxi* or phobia or phobic))).tw,kw. (15988)

46 (incidence* adj5 (somatoform or somatiz* or somatis* or hysteri* or briquet or multisomat* or multi somat* or MUPs or medically unexplained or ((dissociative adj3 (disorder* or reaction*)) or dissociation) or (affective* adj (disorder? or disease? or illness* or symptom?)) or ((stress or cognitive or cognition or personality or impulse control or paranoid or psychotic or neurologic* or nervous or eating) adj (disorder? or illness* or disease?)) or ((bipolar or behavio?ral or obsessive or compulsive or panic or mood or agoraphobi* or phobic or delusional) adj (disorder? or illness* or disease?)) or (OCD or obsess*-compulsi* or GAD or stress reaction? or acute stress or neuros#s or neurotic) or (stress syndrome? or distress syndrome? or pain disorder?) or (depressi* or depressed or MDD or anxi* or phobia or phobic))).tw,kw. (4303)

47 (determinant* adj5 (somatoform or somatiz* or somatis* or hysteri* or briquet or multisomat* or multi somat* or MUPs or medically unexplained or ((dissociative adj3 (disorder* or reaction*)) or dissociation) or (affective* adj (disorder? or disease? or illness* or symptom?)) or ((stress or cognitive or cognition or personality or impulse control or paranoid or psychotic or neurologic* or nervous or eating) adj (disorder? or illness* or disease?)) or ((bipolar or behavio?ral or obsessive or compulsive or panic or mood or agoraphobi* or phobic or delusional) adj (disorder? or illness* or disease?)) or (OCD or obsess*-compulsi* or GAD or stress reaction? or acute stress or neuros#s or neurotic) or (stress syndrome? or distress syndrome? or pain disorder?) or (depressi* or depressed or MDD or anxi* or phobia or phobic))).tw,kw. (901)

48 (cohort* adj5 (somatoform or somatiz* or somatis* or hysteri* or briquet or multisomat* or multi somat* or MUPs or medically unexplained or ((dissociative adj3 (disorder* or reaction*)) or dissociation) or (affective* adj (disorder? or disease? or illness* or symptom?)) or ((stress or cognitive or cognition or personality or impulse control or paranoid or psychotic or neurologic* or nervous or eating) adj (disorder? or illness* or disease?)) or ((bipolar or behavio?ral or obsessive or compulsive or panic or mood or agoraphobi* or phobic or delusional) adj (disorder? or illness* or disease?)) or (OCD or obsess*-compulsi* or GAD or stress reaction? or acute stress or neuros#s or neurotic) or (stress syndrome? or distress syndrome? or pain disorder?) or (depressi* or depressed or MDD or anxi* or phobia or phobic))).tw,kw. (2151)

49 (observational* adj5 (somatoform or somatiz* or somatis* or hysteri* or briquet or multisomat* or multi somat* or MUPs or medically unexplained or ((dissociative adj3 (disorder* or reaction*)) or dissociation) or (affective* adj (disorder? or disease? or illness* or symptom?)) or ((stress or cognitive or cognition or personality or impulse control or paranoid or psychotic or neurologic* or nervous or eating) adj (disorder? or illness* or disease?)) or ((bipolar or behavio?ral or obsessive or compulsive or panic or mood or agoraphobi* or phobic or delusional) adj (disorder? or illness* or disease?)) or (OCD or obsess*-compulsi* or GAD or stress reaction? or acute stress or neuros#s or neurotic) or (stress syndrome? or distress syndrome? or pain disorder?) or (depressi* or depressed or MDD or anxi* or phobia or phobic))).tw,kw. (309)

50 ("risk factor*" adj5 (somatoform or somatiz* or somatis* or hysteri* or briquet or multisomat* or multi somat* or MUPs or medically unexplained or ((dissociative adj3 (disorder* or reaction*)) or dissociation) or (affective* adj (disorder? or disease? or illness* or symptom?)) or ((stress or cognitive or cognition or personality or impulse control or paranoid or psychotic or neurologic* or nervous or eating) adj (disorder? or illness* or disease?)) or ((bipolar or behavio?ral or obsessive or compulsive or panic or mood or agoraphobi* or phobic or delusional) adj (disorder? or illness* or disease?)) or (OCD or obsess*-compulsi* or GAD or stress reaction? or acute stress or neuros#s or neurotic) or (stress syndrome? or distress syndrome? or pain disorder?) or (depressi* or depressed or MDD or anxi* or phobia or phobic))).tw,kw. (7350)

51 or/45-50 [Prevalence or Obs studies of CMDs Textwords] (28554)
52 44 or 51 [Prevalence or Obs studies of CMDs] (130924)
53 8 and 32 and 52 [S Asia NCDs and CMD Prevalence or Obs studies] (608)
54 (somatoform or somatiz* or somatis* or hysteri* or briquet or multisomat* or multi somat* or MUPs or medically unexplained or ((dissociative adj3 (disorder* or reaction*)) or dissociation) or (affective* adj (disorder? or disease? or illness* or symptom?)) or ((stress or cognitive or cognition or personality or impulse control or paranoid or psychotic or neurologic* or nervous or eating) adj (disorder? or illness* or disease?)) or ((bipolar or behavio?ral or obsessive or compulsive or panic or mood or agoraphobi* or phobic or delusional) adj (disorder? or illness* or disease?)) or (OCD or obsess*-compulsi* or GAD or stress reaction? or acute stress or neuros#s or neurotic) or (stress syndrome? or distress syndrome? or pain disorder?) or (depressi* or depressed or MDD or anxi* or phobia or phobic)).tw,kw. (682258)

55 37 or 54 [CMDs] (813547)
56 8 and 32 and 55 (2590)
57 limit 56 to systematic reviews (70)
58 53 or 57 [S Asia NCDs and CMD Prevalence or Obs studies or Systematic Reviews] (658)
59 limit 58 to yr="1990 -Current" (653)

60 animals/ not humans/ (4433348)
61 (exp Child/ or Adolescent/ or exp Infant/) not exp Adult/ (1747799)
62 59 not (60 or 61) [Final search animals and children removed] (608)

Ovid MEDLINE(R) In-Process & Other Non-Indexed Citations <July 02, 2018>
1 ((Indian or Indians) not ("west indian*" or "american indian*")).ti,ab,in. (25566)
2 india.ti,ab,in. (137980)
3 ("south asia*" or bangladesh* or pakistan*).ti,ab,in. (13194)
4 (Bhutan* or afghanistan* or maldive* or nepal* or "sri lanka*").ti,ab,in. (4718)
5 exp south asia/ (0)
6 or/1-5 [All S Asia World Bank] (157513)
7 (co-morbid* or comorbid*).ti,ab. (19607)
8 (multi-morbid* or multimorbid*).ti,ab. (683)
9 (("long term" or chronic) adj3 (disease? or illness* or condition? or disorder?)).tw. (34094)
10 diabet*.tw. (56399)
11 (Aneurysm* or Atherosclerosis or Cardio* or endocard* or pericard*).tw. (80728)
12 (heart or cardiac or valvular or coronary or arterial or vascular or hypertension).tw. (154207)
13 ((long-term or chronic*) adj2 (airway* or pulmonar* or airflow* or lung*) adj2 Obstruct*).tw. (4898)
14 (pulmonar* adj2 emphysem*).tw. (154)
15 ((long-term or chronic*) adj5 (bronchitis or respirat*)).tw. (1480)
16 ((long-term or Chronic*) adj2 lung* adj5 (condition* or disease* or symptom* or problem* or failure*)).tw. (949)
17 (respiratory adj2 (condition* or disease* or symptom* or problem*)).tw. (4295)
18 (stroke or poststroke or post-stroke or cerebrovasc* or brain vasc* or cerebral vasc* or apoplex*).tw. (26659)
19 ((brain? or cerebr* or cerebell* or intracran* or intracerebral) adj2 (isch?emi* or infarct* or thrombo* or emboli* or occlus*)).tw. (6454)
20 ((brain? or cerebr* or cerebell* or intracerebral or intracranial or subarachnoid) adj2 (h?emorrhage? or h?ematoma* or bleed*)).tw. (5284)
21 (transi* adj3 isch?em* adj3 attack?).tw. (1160)
22 (cancer* or carcinoma? or tumor? or tumour? or neoplasm? or malignan*).tw. (264401)
23 or/7-22 [Chronic or Multimorbid incl Cancer] (521202)
24 (somatoform or somatiz* or somatis* or hysteri* or briquet or multisomat* or multi somat* or MUPs or medically unexplained).tw. (1228)
25 ((dissociative adj3 (disorder* or reaction*)) or dissociation).tw. (14258)
26 (affective* adj (disorder? or disease? or illness* or symptom?)).tw. (1197)
27 ((stress or cognitive or cognition or personality or impulse control or mood or paranoid or psychotic or neurologic* or nervous or eating) adj (disorder? or illness* or disease?)).tw. (14655)
28 ((bipolar or behavio?ral or obsessive or compulsive or panic or mood or agoraphobi* or delusional) adj (disorder? or illness* or disease?)).tw. (6652)
29 (OCD or obsess*-compulsi* or GAD or stress reaction? or acute stress or neuros#s or neurotic).tw. (3941)
30 (stress syndrome? or distress syndrome? or pain disorder?).tw. (2410)
31 (depressi* or depressed or MDD or anxi* or phobia or phobic).tw. (45512)
32 (stress adj5 (chronic* or long-term or severe or mental* or psyc*)).ab. (4988)
33 stress.ti. (21762)
34 or/24-33 [CMDs] (97847)
35 6 and 23 and 34 [S Asia NCDs CMDs] (1401)
36 limit 35 to systematic reviews (23)
37 (incidence* adj5 (somatoform or somatiz* or somatis* or hysteri* or briquet or multisomat* or multi somat* or MUPs or medically unexplained or ((dissociative adj3 (disorder* or reaction*)) or dissociation) or (affective* adj (disorder? or disease? or illness* or symptom?)) or ((stress or cognitive or cognition or personality or impulse control or paranoid or psychotic or neurologic* or nervous or eating) adj (disorder? or illness* or disease?)) or ((bipolar or behavio?ral or obsessive or compulsive or panic or mood or agoraphobi* or phobic or delusional) adj (disorder? or illness* or disease?)) or (OCD or obsess*-compulsi* or GAD or stress reaction? or acute stress or neuros#s or neurotic) or (stress syndrome? or distress syndrome? or pain disorder?) or (depressi* or depressed or MDD or anxi* or phobia or phobic))).tw,kw. (419)

38 (prevalen* adj5 (somatoform or somatiz* or somatis* or hysteri* or briquet or multisomat* or multi somat* or MUPs or medically unexplained or ((dissociative adj3 (disorder* or reaction*)) or dissociation) or (affective* adj (disorder? or disease? or illness* or symptom?)) or ((stress or cognitive or cognition or

personality or impulse control or paranoid or psychotic or neurologic* or nervous or eating) adj (disorder? or illness* or disease?)) or ((bipolar or behavio?ral or obsessive or compulsive or panic or mood or agoraphobi* or phobic or delusional) adj (disorder? or illness* or disease?)) or (OCD or obsess*-compulsi* or GAD or stress reaction? or acute stress or neuros#s or neurotic) or (stress syndrome? or distress syndrome? or pain disorder?) or (depressi* or depressed or MDD or anxi* or phobia or phobic))).tw,kw. (2377)

39 (determinant* adj5 (somatoform or somatiz* or somatis* or hysteri* or briquet or multisomat* or multi somat* or MUPs or medically unexplained or ((dissociative adj3 (disorder* or reaction*)) or dissociation) or (affective* adj (disorder? or disease? or illness* or symptom?)) or ((stress or cognitive or cognition or personality or impulse control or paranoid or psychotic or neurologic* or nervous or eating) adj (disorder? or illness* or disease?)) or ((bipolar or behavio?ral or obsessive or compulsive or panic or mood or agoraphobi* or phobic or delusional) adj (disorder? or illness* or disease?)) or (OCD or obsess*-compulsi* or GAD or stress reaction? or acute stress or neuros#s or neurotic) or (stress syndrome? or distress syndrome? or pain disorder?) or (depressi* or depressed or MDD or anxi* or phobia or phobic))).tw,kw. (122)

40 (cohort* adj5 (somatoform or somatiz* or somatis* or hysteri* or briquet or multisomat* or multi somat* or MUPs or medically unexplained or ((dissociative adj3 (disorder* or reaction*)) or dissociation) or (affective* adj (disorder? or disease? or illness* or symptom?)) or ((stress or cognitive or cognition or personality or impulse control or paranoid or psychotic or neurologic* or nervous or eating) adj (disorder? or illness* or disease?)) or ((bipolar or behavio?ral or obsessive or compulsive or panic or mood or agoraphobi* or phobic or delusional) adj (disorder? or illness* or disease?)) or (OCD or obsess*-compulsi* or GAD or stress reaction? or acute stress or neuros#s or neurotic) or (stress syndrome? or distress syndrome? or pain disorder?) or (depressi* or depressed or MDD or anxi* or phobia or phobic))).tw,kw. (305)

41 (observational* adj5 (somatoform or somatiz* or somatis* or hysteri* or briquet or multisomat* or multi somat* or MUPs or medically unexplained or ((dissociative adj3 (disorder* or reaction*)) or dissociation) or (affective* adj (disorder? or disease? or illness* or symptom?)) or ((stress or cognitive or cognition or personality or impulse control or paranoid or psychotic or neurologic* or nervous or eating) adj (disorder? or illness* or disease?)) or ((bipolar or behavio?ral or obsessive or compulsive or panic or mood or agoraphobi* or phobic or delusional) adj (disorder? or illness* or disease?)) or (OCD or obsess*-compulsi* or GAD or stress reaction? or acute stress or neuros#s or neurotic) or (stress syndrome? or distress syndrome? or pain disorder?) or (depressi* or depressed or MDD or anxi* or phobia or phobic))).tw,kw. (69)

42 ("risk factor*" adj5 (somatoform or somatiz* or somatis* or hysteri* or briquet or multisomat* or multi somat* or MUPs or medically unexplained or ((dissociative adj3 (disorder* or reaction*)) or dissociation) or (affective* adj (disorder? or disease? or illness* or symptom?)) or ((stress or cognitive or cognition or personality or impulse control or paranoid or psychotic or neurologic* or nervous or eating) adj (disorder? or illness* or disease?)) or ((bipolar or behavio?ral or obsessive or compulsive or panic or mood or agoraphobi* or phobic or delusional) adj (disorder? or illness* or disease?)) or (OCD or obsess*-compulsi* or GAD or stress reaction? or acute stress or neuros#s or neurotic) or (stress syndrome? or distress syndrome? or pain disorder?) or (depressi* or depressed or MDD or anxi* or phobia or phobic))).tw,kw. (1005)

43 or/37-42 [CMD prevalence or obs studies] (3909)
44 6 and 23 and 43 [S Asia NCD CMD prevalence or obs studies] (112)
45 36 or 44 [S Asia NCD CMD prevalence or obs studies or systematic reviews] (131)
46 limit 45 to yr="1990 -Current" (131)
47 (rat or rats or mice or mouse).ti. (54182)
48 ((child* or infant* or baby or babies or preterm* or adolesc* or newborn* or pediatric* or paediatri* or neonate* or teen* or schoolchild*) not (adult or elderly or geriatric*)).ti. (90309)
49 46 not (47 or 48) [Final search animals and children removed] (119)

Ovid MEDLINE(R) Epub Ahead of Print <July 02, 2018>
Same search strategy as Ovid MEDLINE(R) In-Process & Other Non-Indexed Citations <July 02, 2018>

PakMediNet (PakCyber) Inception – 13-09-2018
107 brief searches were run using 'Simple' search interface as 'Advanced' interface was not functioning. The terms entered are automatically combined with an AND Boolean operator, for example searching: depression cancer prevalence runs a search for depression AND cancer AND prevalence

Searches for Cancer or Stroke	Searches for Cardiovascular Diseases	Searches for COPD or Diabetes
depression cancer prevalence	depression cardiovascular prevalence	depression pulmonary prevalence
depression cancer epidemiology	depression cardiovascular epidemiology	depression pulmonary incidence
depression cancer incidence	depression cardiovascular incidence	depression pulmonary observational
depression cancer observational	depression cardiovascular observational	anxiety pulmonary
anxiety cancer prevalence	anxiety cardiovascular prevalence	stress pulmonary incidence
anxiety cancer epidemiology	anxiety cardiovascular epidemiology	stress pulmonary prevalence
anxiety cancer incidence	anxiety cardiovascular incidence	stress pulmonary epidemiology
anxiety cancer observational	anxiety cardiovascular observational	stress pulmonary observational
stress cancer prevalence	stress cardiovascular prevalence .	distress pulmonary prevalence
stress cancer epidemiology	Stress cardiovascular epidemiology	distress pulmonary epidemiology
stress cancer incidence	stress cardiovascular incidence	distress pulmonary incidence
stress cancer observational	Stress cardiovascular observational	distress pulmonary observational
distress cancer	distress cardiovascular prevalence	Phobia pulmonary
Phobia cancer	distress cardiovascular incidence	Affective pulmonary
affective cancer	distress cardiovascular observational	somatoform pulmonary
somatoform cancer	Distress cardiovascular epidemiology	behavioral pulmonary
behavioural cancer	Phobia cardiovascular	behavioural pulmonary
behavioral cancer	affective cardiovascular	cognitive pulmonary
Panic cancer	somatoform cardiovascular	Panic pulmonary
Compulsive cancer	behavioural cardiovascular	Compulsive pulmonary
cognitive cancer	behavioral cardiovascular	depression diabetes prevalence
depression stroke prevalence	Panic cardiovascular	Depression Diabetes Epidemiology
Depression stroke epidemiology	compulsive cardiovascular	depression diabetes incidence
Depression stroke incidence	cognitive cardiovascular	depression diabetes observational
depression stroke observational		anxiety diabetes prevalence
anxiety stroke		anxiety diabetes incidence
stress stroke prevalence		anxiety diabetes observational
Stress stroke epidemiology		Anxiety Diabetes Epidemiology
stress stroke incidence		stress diabetes prevalence
stress stroke observational		Stress diabetes epidemiology
distress stroke		stress diabetes incidence
Phobia stroke		stress diabetes observational
affective stroke		distress diabetes prevalence
behavioural stroke		Distress diabetes epidemiology
behavioral stroke		distress diabetes incidence
panic stroke		distress diabetes observational
compulsive stroke		phobia diabetes
cognitive stroke		affective diabetes
		somatoform diabetes
		behavioural diabetes
		behavioral diabetes
		Panic diabetes
		Compulsive diabetes
		cognitive diabetes

PsycINFO 1806 to June Week 4 2018

1 ((Indian or Indians) not ("west indian*" or "american indian*")).ti,ab,in,id,lo. (16682)
2 india.ti,ab,in,id,lo. (38957)
3 ("south asia*" or bangladesh* or pakistan*).ti,ab,in,id,lo. (8855)
4 (Bhutan* or afghanistan* or maldive* or nepal* or "sri lanka*").ti,ab,in,id,lo. (6452)
5 or/1-4 [All S Asia World Bank] (57550)
6 comorbidity/ (28957)
7 (co-morbid* or comorbid*).ti,ab,id. (53103)
8 (multi-morbid* or multimorbid*).ti,ab,id. (786)

9 Chronic illness/ (10638)
10 (("long term" or chronic) adj3 (disease? or illness* or condition? or disorder?)).tw,id. (43120)
11 exp diabetes/ (15988)
12 exp Cardiovascular Disorders/ (57443)
13 exp Chronic Obstructive Pulmonary Disease/ (1240)
14 exp Cerebrovascular Accidents/ (19139)
15 exp Neoplasms/ (47053)
16 diabet*.tw,id. (28538)
17 (Aneurysm* or Atherosclerosis or Cardio* or endocard* or pericard*).tw,id. (37682)
18 ((long-term or chronic*) adj2 (airway* or pulmonar* or airflow* or lung*) adj2 Obstruct*).tw,id. (2180)
19 (pulmonar* adj2 emphysem*).tw,id. (18)
20 ((long-term or chronic*) adj5 (bronchitis or respirat*)).tw,id. (649)
21 ((long-term or Chronic*) adj2 lung* adj5 (condition* or disease* or symptom* or problem* or failure*)).tw,id. (367)
22 (respiratory adj2 (condition* or disease* or symptom* or problem*)).tw,id. (1929)
23 (stroke or poststroke or post-stroke or cerebrovasc* or brain vasc* or cerebral vasc* or apoplex*).tw,id. (35772)
24 ((brain? or cerebr* or cerebell* or intracran* or intracerebral) adj2 (isch?emi* or infarct* or thrombo* or emboli* or occlus*)).tw,id. (7518)
25 ((brain? or cerebr* or cerebell* or intracerebral or intracranial or subarachnoid) adj2 (h?emorrhage? or h?ematoma* or bleed*)).tw,id. (4130)
26 (transi* adj3 isch?em* adj3 attack?).tw,id. (1110)
27 (cancer* or carcinoma? or tumor? or tumour? or neoplasm? or malignan*).tw,id. (72693)
28 or/6-27 [Multimorbidity or Chronic Non Communicable disease] (271989)
29 anxiety disorders/ or acute stress disorder/ or castration anxiety/ or death anxiety/ or generalized anxiety disorder/ or exp obsessive compulsive disorder/ or panic disorder/ or exp phobias/ or separation anxiety disorder/ (48708)
30 major depression/ or anaclitic depression/ or endogenous depression/ or late life depression/ or postpartum depression/ or reactive depression/ or recurrent depression/ or treatment resistant depression/ (117913)
31 exp stress/ (99632)
32 (somatoform or somatiz* or somatis* or hysteri* or briquet or multisomat* or multi somat* or MUPs or medically unexplained).tw,id. (16132)
33 ((dissociative adj3 (disorder* or reaction*)) or dissociation).tw,id. (19307)
34 (affective* adj (disorder? or disease? or illness* or symptom?)).tw,id. (19480)
35 ((stress or cognitive or cognition or personality or impulse control or mood or paranoid or psychotic or neurologic* or nervous or eating) adj (disorder? or illness* or disease?)).tw,id. (125076)
36 ((bipolar or behavio?ral or obsessive or compulsive or panic or mood or agoraphobi* or delusional) adj (disorder? or illness* or disease?)).tw,id. (67655)
37 (OCD or obsess*-compulsi* or GAD or stress reaction? or acute stress or neuros#s or neurotic).tw,id. (55900)
38 (stress syndrome? or distress syndrome? or pain disorder?).tw,id. (2090)
39 (depressi* or depressed or MDD or anxi* or phobia or phobic).tw,id. (401075)
40 (stress adj5 (chronic* or long-term or severe or mental* or psyc*)).ab. (41105)
41 stress.ti. (67580)
42 or/29-41 [CMDs] (650805)
43 5 and 28 and 42 [S Asia CMDs and Multimorbidity or Non Communicable Disease] (1114)
44 limit 43 to "reviews (maximizes specificity)" (35)
45 epidemiology/ (46998)
46 at risk populations/ (35803)
47 risk factors/ (70989)
48 cohort analysis/ or followup studies/ or exp longitudinal studies/ (29324)
49 prevalen*.tw,id. (130118)
50 incidence*.tw,id. (48885)
51 epidemiolog*.tw,id. (48917)
52 risk factor*.tw,id. (94276)
53 ((study or studies) adj3 (case-control or cohort studies or follow-up or follow-on or longitudinal or prospective or retrospective or cross-sectional)).tw,id. (138795)
54 or/45-53 [Prevleance or Obs Studies] (435905)
55 43 and 54 [S Asia NCD CMD Prevalence] (441)
56 44 or 55 [S Asia NCD CMD Prevalence Obs Studies or Systematic reviews] (453)

57 limit 56 to yr="1990 -Current" (442)
58 ((child* or adolescen*) not adult*).ag. (476759)
59 (rat or rats or mouse or mice).ti. (111207)
60 57 not (58 or 59) [Final search animals and children removed] (414)

World Bank Group Research and Publications: Documents and Reports
http://documents.worldbank.org/curated/en/docadvancesearch/ (accessed 13-09-2018)
Six searches run due to technical issues with executing searches with long search strings

1. (depression OR depressed OR depressive OR anxiety OR stress OR distress OR phobia OR affective) searched in Keywords Field, 'Boolean query' selected.

Filters applied:

Country - Bangladesh, India, Pakistan

Topic - Health, Nutrition and Population

Document type: Publications & Research

Search, and then tick 'Search within' to limit search

(cancer OR neoplasm OR diabetes) searched within Search Documents field

2. (depression OR depressed OR depressive OR anxiety OR stress OR distress OR phobia OR affective) searched in Keywords Field, 'Boolean query' selected.

Filters applied: same as search 1

Search, and then tick 'Search within' to limit search

(stroke OR cardiovascular OR vascular OR cardiac OR coronary OR arterial OR hypertension) searched within Search Documents field

3. (depression OR depressed OR depressive OR anxiety OR stress OR distress OR phobia OR affective) searched in Keywords Field, 'Boolean query' selected.

Filters applied: same as search 1

Search, and then tick 'Search within' to limit search

(pulmonary OR lung OR respiratory OR bronchitis OR Emphysema) searched within Search Documents field

4. (somatoform OR behavioral OR behavioural OR panic OR compulsive OR cognitive) searched in Keywords Field, 'Boolean query' selected.

Filters applied: same as search 1

Search, and then tick 'Search within' to limit search

(cancer OR neoplasm OR diabetes) searched within Search Documents field

5. (somatoform OR behavioral OR behavioural OR panic OR compulsive OR cognitive) searched in Keywords Field, 'Boolean query' selected.

Filters applied: same as search 1

Search, and then tick 'Search within' to limit search

(stroke OR cardiovascular OR vascular OR cardiac OR coronary OR arterial OR hypertension) searched within Search Documents field

6. (somatoform OR behavioral OR behavioural OR panic OR compulsive OR cognitive) searched in Keywords Field, 'Boolean query' selected.

Filters applied: same as search 1

Search, and then tick 'Search within' to limit search

(pulmonary OR lung OR respiratory OR bronchitis OR Emphysema) searched within Search Documents field

Figure S1. Overview of quality assessment

Legend:

AXIS tool items: 1 aims/objectives, 2 study design, 3 sample size, 4 target population, 5 sample frame, 6 selection process, 7 non-responders, 8 outcome measures, 9 instruments, 10 statistical significance, 11 description of methods, 12 description basic data, 13 response rate, 14 description non-responders, 15 consistency results, 16 analyses, 17 discussion and conclusions, 18 study limitations, 19 conflicts of interest, 20 ethical approval.

To calculate summary scores, studies received 1 point for a green box, 0.5 point for an orange box, and no points for a red box. All items were weighted equally.

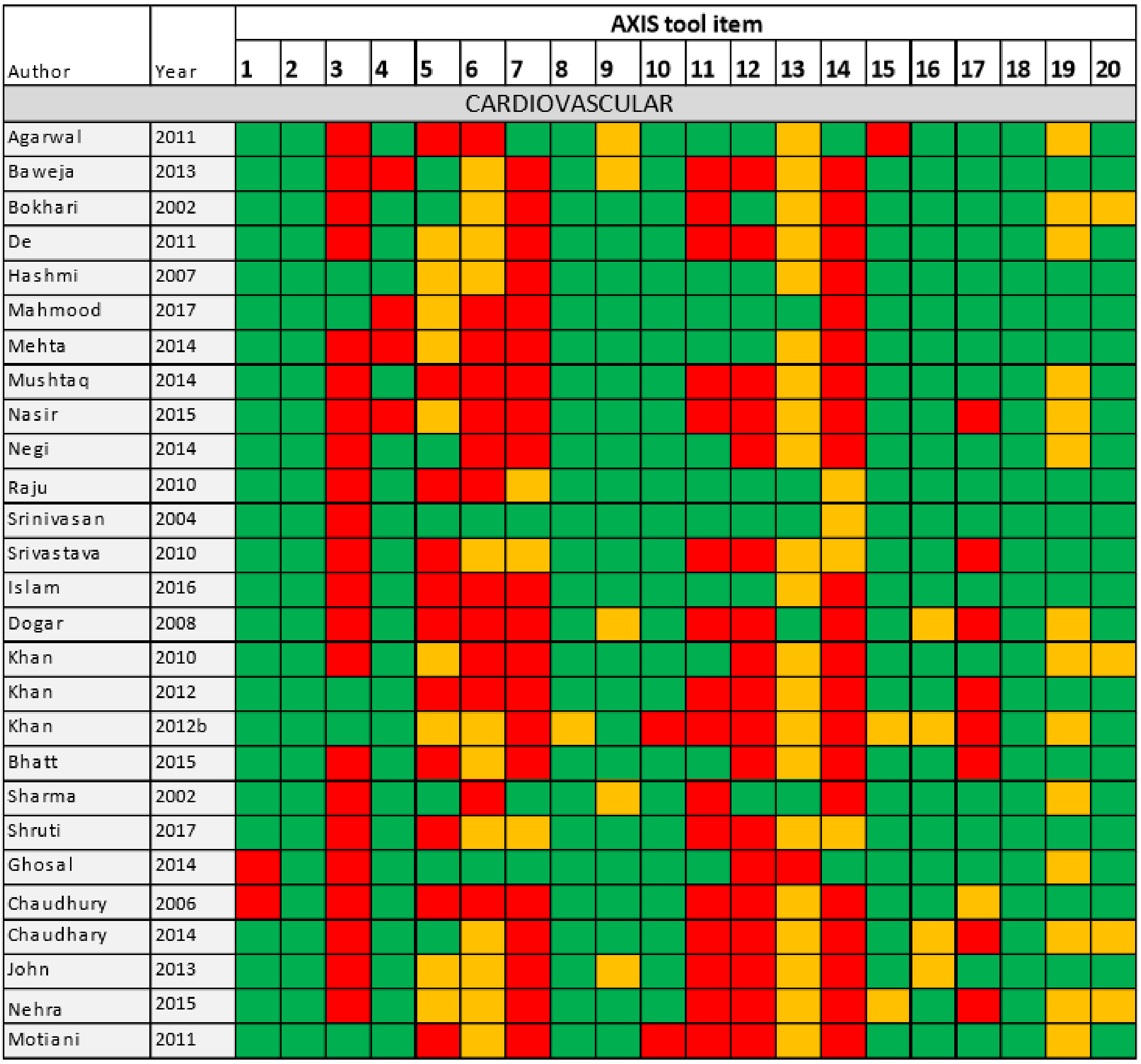

Author	Year	AXIS tool item																			
		1	2	3	4	5	6	7	8	9	10	11	12	13	14	15	16	17	18	19	20
CANCER																					
Chaitanya	2016	Green	Green	Red	Green	Green	Green	Red	Green	Green	Green	Red	Red	Yellow	Red	Green	Green	Green	Green	Green	Green
Mishra	2006	Green	Green	Red	Green	Red	Yellow	Red	Green	Yellow	Green	Green	Red	Red	Red	Green	Green	Green	Red	Green	Green
Mohanti	2015	Green	Green	Red	Green	Red	Red	Red	Green	Green	Red	Red	Red	Yellow	Red	Green	Green	Green	Green	Yellow	Green
Pandey	2007	Green	Green	Red	Red	Yellow	Yellow	Red	Green	Green	Green	Red	Red	Red	Red	Yellow	Green	Green	Red	Yellow	Green
Purkayastha	2017	Green	Green	Green	Green	Red	Red	Green	Green	Green	Green	Green	Green	Yellow	Red	Green	Green	Red	Green	Green	Green
Rashid	2012	Green	Green	Green	Green	Red	Yellow	Red	Green	Green	Red	Green	Green	Yellow	Red	Green	Green	Green	Red	Yellow	Green
Sutanay	2017	Green	Green	Green	Green	Green	Green	Red	Green	Green	Green	Green	Green	Yellow	Red	Green	Green	Green	Green	Green	Green
Alexander	1993	Green	Green	Red	Green	Red	Red	Green	Green	Green	Green	Green	Red	Yellow	Red	Green	Green	Green	Green	Yellow	Red
Chandra	1998	Green	Green	Red	Green	Green	Red	Red	Green	Green	Green	Red	Red	Yellow	Red	Green	Green	Green	Green	Yellow	Green
Jadoon	2010	Green	Green	Red	Green	Red	Red	Green	Green	Green	Green	Green	Green	Green	Red	Red	Green	Green	Green	Green	Green
Khalil	2016	Green	Green	Green	Green	Green	Yellow	Green	Green	Green	Green	Red	Green	Green	Green	Green	Green	Green	Green	Green	Green
Nausheen	2007	Green	Green	Red	Green	Green	Yellow	Red	Green	Green	Green	Red	Green	Yellow	Red	Green	Green	Green	Green	Yellow	Green
Chaturvedi	1996	Green	Green	Red	Green	Red	Red	Red	Green	Green	Green	Red	Red	Yellow	Yellow	Yellow	Green	Red	Red	Yellow	Yellow
Chawla	1999	Green	Green	Red	Green	Yellow	Yellow	Red	Green	Green	Green	Red	Red	Yellow	Red	Green	Green	Green	Red	Yellow	Green
Chittem	2013	Green	Green	Red	Green	Red	Red	Green	Green	Green	Green	Green	Green	Green	Green	Green	Green	Green	Green	Yellow	Green
Deshpande	2014	Green	Green	Red	Green	Green	Yellow	Red	Green	Green	Green	Green	Red	Yellow	Red	Green	Green	Green	Green	Yellow	Green
Jadhav	2010	Red	Green	Red	Green	Red	Yellow	Green	Green	Green	Green	Red	Red	Green	Green	Yellow	Yellow	Red	Red	Green	Green
Kausar	2000	Green	Green	Red	Green	Red	Yellow	Green	Green	Green	Red	Red	Red	Red	Red	Yellow	Yellow	Green	Green	Yellow	Yellow
Thomas	2005	Green	Green	Red	Green	Green	Yellow	Red	Green	Yellow	Green	Red	Red	Yellow	Red	Yellow	Green	Green	Red	Yellow	Green
Mendonsa	2010	Green	Green	Red	Green	Green	Red	Red	Green	Green	Green	Green	Yellow	Yellow	Red	Green	Green	Red	Green	Green	Green
Hashmi	2013	Green	Green	Red	Green	Yellow	Yellow	Green	Green	Green	Green	Red	Red	Yellow	Red	Green	Green	Green	Green	Green	Green
Shankar	2016	Green	Green	Red	Green	Green	Yellow	Red	Green	Green	Green	Red	Red	Yellow	Red	Green	Yellow	Green	Green	Green	Green
Ahmad	2009	Green	Green	Red	Green	Green	Yellow	Red	Green	Green	Red	Red	Red	Yellow	Red	Yellow	Green	Red	Green	Yellow	Green

Author	Year	AXIS tool item																			
		1	2	3	4	5	6	7	8	9	10	11	12	13	14	15	16	17	18	19	20
DIABETES																					
Ali	2013																				
Arshad	2016																				
Bajaj	2012																				
Das	2013																				
Farhan	2017																				
Joseph	2013																				
Khan	2014																				
Khullar	2016																				
Khuwaja	2010																				
Madhu	2013																				
Mathew	2012																				
Mushtaque	2016																				
Natasha	2015																				
Poongothai	2011																				
Rajput	2016																				
Raval	2010																				
Roy	2012																				
Thour	2015																				
Weaver	2015																				
Zahid	2008																				
Zuberi	2011																				
Azad	2014																				
Balhara	2011																				
Chaudhary	2017																				
Chaudhry	2010																				
Ingle	2017																				
Iype	2009																				
Jain	2015																				
Lloyd	2018																				
Mir	2015																				
Bahety	2017																				
Islam	2015																				
Perveen	2010																				
Siddiqui	2014																				
Singh	2014																				
Asghar	2007																				
Gehlawat	2013																				
Rauf	2016																				
Rauf	2005																				
Siddique	2014																				

Author	Year	AXIS tool item																			
		1	2	3	4	5	6	7	8	9	10	11	12	13	14	15	16	17	18	19	20
RESPIRATORY																					
De	2012																				
Godil	2017																				
Agarwal	2018																				
Misra	2015																				
MULTIPLE CONDITIONS																					
Buvneshkumar	2018																				

Figure S2. Funnel plot of logit depression prevalence estimates against sample size

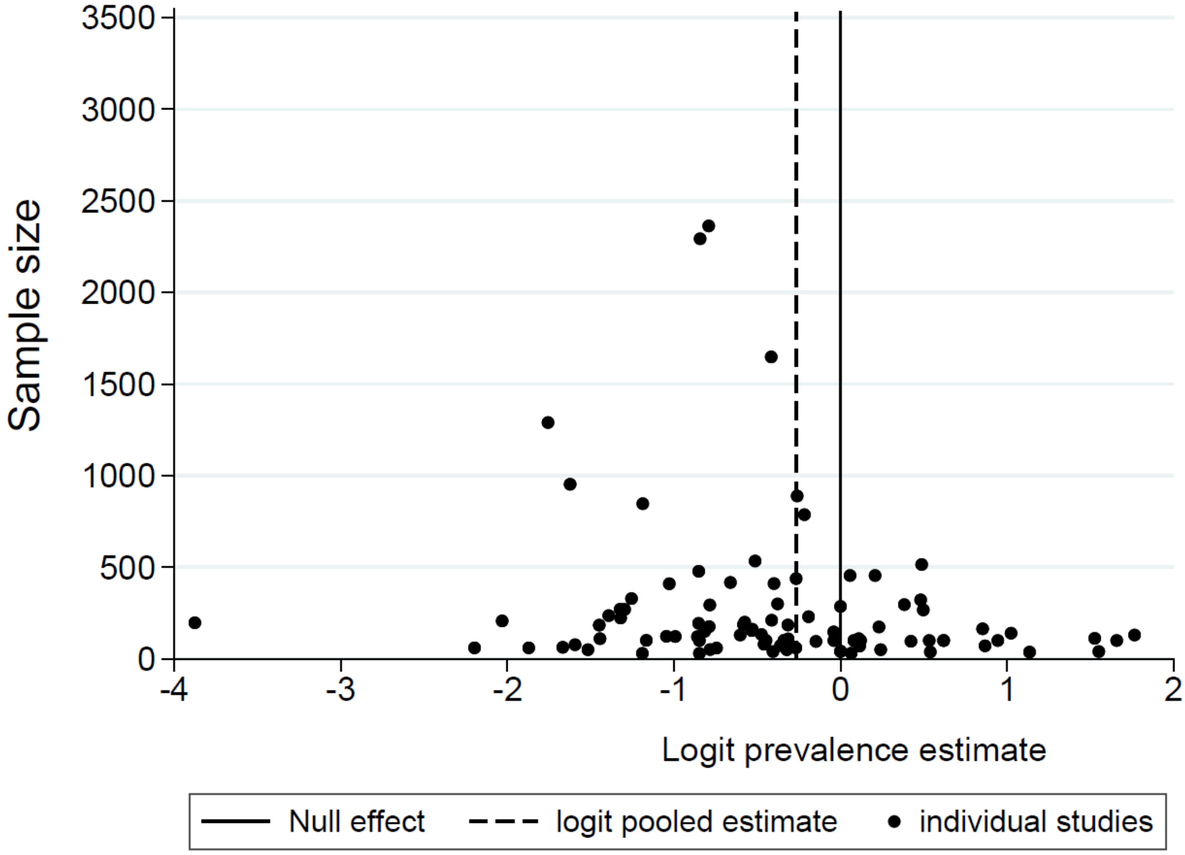

Table S1. Meta regression analysis depression prevalence

Prevalence of depression	N=94, tau2=0·02, I^2 res=83%, R^2 adj=0·42%		
**Variable**	**β coefficient**	**SE**	**95% CI**
Year of publication	0·01	0·004	-0·001; 0·016
Sample size (vs ≤ 50)			
51-100	-0·02	0·077	-0·173 to 0·133
101-300	-0·09	0·073	-0·232 to 0·060
>300	-0·10	0·080	-0·266 to 0·054
CMD diagnostic tool (vs other)			
diagnostic interview	-0·04	0·069	-0·176 to 0·100
Quality score[1]	-0·01	0·010	-0·176 to 0·008
Country			
Bangladesh	-0·02	0·078	-0·171 to 0·138
Pakistan	-0·02	0·081	-0·184 to 0·137
NCD			
Cancer	-0·00	0·055	-0·112 to 0·109
CVD	0·05	0·048	-0·040 to 0·150
Respiratory	0·03	0·073	-0·114 to 0·177

1. A higher score indicating better quality

Table S2. Meta regression analysis anxiety prevalence

Prevalence of anxiety	N=28, tau2 = 0·02, I^2 res=80%, R^2 adj=11%		
**Variable**	**β coefficient**	**SE**	**95% CI**
Year of publication	0·01	0·006	-0·005 to 0·022
Sample size (vs ≤ 50)			
51-100	0·04	0·106	-0·177 to 0·264
101-300	0·06	0·102	-0·149 to 0·276
>300	0·21	0·120	-0·036 to 0·463
Quality score[1]	-0·03	0·020	-0·071 to 0·013

1. A higher score indicating better quality

Appendix S2. Full reference list of included studies

1. Nasir U, Shahid H, Shabbir MO. Sleep quality and depression in hospitalized congestive heart failure patients. JPMA - J Pakistan Med Assoc. 2015;65:264–9.

2. Dogar IA, Khawaja IS, Azeem MW, Awan H, Ayub A, Iqbal J, et al. Prevalence and risk factors for depression and anxiety in hospitalized cardiac patients in pakistan. Psychiatry. 2008;5:38–41.

3. Khan FH, Ambreen K, Fatima G, Kumar S. Assessment of health risks with reference to oxidative stress and DNA damage in chromium exposed population. Sci Total Environ. 2012;430:68–74.

4. Chaudhury S, Sharma S, Pawar AA, Kumar BK, Srivastava MK, Sudarsanan S, et al. Psychological Correlates of Outcome after Coronary Artery Bypass Graft. Med J Armed Forces India [Internet]. 2006;62:220–3. Available from: https://www.ncbi.nlm.nih.gov/pmc/articles/PMC4922908/

5. Nehra DK, Sharma NR, Ali G, Margoob MA, Mushtaq H, Kumar P, et al. Comparative Study of Prevalence of Psychological Distress Factors in Coronary Heart Disease Patients Living Under Disturbed Conditions and a normal place of North India. 2015; Available from: http://medind.nic.in/daa/t12/i1/daat12i1p99.pdf

6. A CRKPWGMBS. Psychiatric manifestations maong cardiac patients: A hospital based study. Delhi Psychiatry Journal 2014 oct; 17(2) 253-57 [Internet]. Available from: http://medind.nic.in/daa/t14/i2/daat14i2p253.pdf

7. Mushtaq M, Najam N. Depression, anxiety, stress and demographic determinants of hypertension disease. Pakistan J Med Sci [Internet]. 2014;30:1293–8. Available from: https://www.ncbi.nlm.nih.gov/pmc/articles/PMC4320718/

8. John S. Prevalence and pattern of psychiatric morbidity and health related quality of life in patients with ischemic heart disease in a tertiary care hospital. Indian J Psychiatry 2013 Oct; 55(4) 353-9 [Internet]. Available from: https://www.ncbi.nlm.nih.gov/pmc/articles/PMC3890914/

9. Baweja R, Avasthi A, Chakrabarti S, Prabhakar S. Psychiatric morbidity in patients with transverse myelitis and stroke: A comparison. Indian J Psychiatry [Internet]. 2013;55:59–62. Available from: https://www.ncbi.nlm.nih.gov/pmc/articles/PMC3574457/

10. Khan S, Khan A, Ghaffar R, Awan ZA. Frequency of depression in patients with chronic heart failure. J Ayub Med Coll Abbottabad JAMC. 2012;24:26–9.

11. Motiani B, Haidri FR, Rizvi N. Frequency of depression in Chronic Obstructive Pulmonary Disease (COPD) patients. 2011;27(5):2–5.

12. De S. Prevalence of depression in stable chronic obstructive pulmonary disease. Indian J Chest Dis Allied Sci. 2011;53(8):1–5.

13. Raka S, Karuna J, Vidyasagar D, Velaphi S, Bhat VB. Incidences of hypertension in teachers of JNV University, Jodhpur Surfactant replacement therapy in developing countries. Adv Res J Soc Sci. 2010;1:149–51.

14. Khan MA, Karamat M, Hafizullah M, Nazar Z, Fahim M, Gul AM. Frequency of anxiety and psychosocial stressful events in patients with acute myocardial infraction. J Ayub Med Coll Abbottabad JAMC. 2010;22:32–5.

15. Bhatt P, Parikh P, Patel A, Parikh R, Patel A, Mehta JL, et al. Unique Aspects of Coronary Artery Disease in Indian Women. Cardiovasc Drugs Ther [Internet]. 2015;29:369–76. Available from: https://link.springer.com/article/10.1007%2Fs10557-015-6594-5

16. Bokhari SS, Samad AH, Hanif S, Hadique S, Cheema MQ, Fazal MA, et al. Prevalence of depression in patients with coronary artery disease in a tertiary care hospital in Pakistan. JPMA - J Pakistan Med Assoc [Internet]. 2002;52:436–9. Available from: http://jpma.org.pk/full_article_text.php?article_id=2417

17. Mehta JR, Ratnani IJ, Dave JD, Panchal BN, Patel AK, Vala AU. Association of psychiatric co-morbidities and quality of life with severity of chronic obstructive pulmonary disease. East Asian Arch Psychiatry [Internet]. 2014;24:148–55. Available from: http://www.easap.asia/index.php/component/k2/item/96-1404-v24n4-p148

18. Negi H, Sarkar M, Raval ADD, Pandey K, Das P. Presence of depression and its risk factors in patients with chronic obstructive pulmonary disease. Indian J Med Res. 2014;139(MAR):402–8.

19. Shruti S, Skand S, Manjeet B, Shridhar D. Quality of life in patients with coronary artery disease and panic disorder: a comparative study. Oman Med J. 2017;32(1):20–6.

20. Agarwal M, Trivedi JK, Sinh PK, Dalal PK, Saran RK. Depression in patients of myocardial infarction--a cross-sectional study in northern India. J Assoc Physicians India [Internet]. 2011;59(october):636–638,643. Available from: http://www.ncbi.nlm.nih.gov/pubmed/22479743

21. Ariful Islam M, Rahman A, Aleem MA, Islam SMS. Prevalence and associated factors of depression among post-stroke patients in Bangladesh. Int J Ment Health Addict. 2016;14:154–66.

22. Sharma P, Avasthi A, Chakrabarti S, Varma S. Depression among hospitalised medically ill patients: a two-stage screening study. J Affect Disord. 2002;70:205–9.

23. Mahmood S, Hassan SZ, Tabraze M, Khan MO, Javed I, Ahmed A, et al. Prevalence and Predictors of Depression Amongst Hypertensive Individuals in Karachi, Pakistan. Cureus [Internet]. 2017;9:e1397. Available from: https://www.ncbi.nlm.nih.gov/pmc/articles/PMC5572043/

24. Ghosal MK, Burman P, Singh V, Das S, Paul N, Ray BK, et al. Correlates of functional outcome among stroke survivors in a developing country--a prospective community-based study from India. J Stroke Cerebrovasc Dis. 2014;23:2614–21.

25. Raju RS, Sarma PS, Pandian JD. Psychosocial problems, quality of life, and functional independence among Indian stroke survivors. Stroke. 2010;41:2932–7.

26. Hashmi SK, Afridi MB, Abbas K, Sajwani RA, Saleheen D, Frossard PM, et al. Factors associated with adherence to anti-hypertensive treatment in Pakistan. PLoS ONE [Electronic Resour. 2007;2:e280.

27. Srinivasan K, Joseph W. A study of lifetime prevalence of anxiety and depressive disorders in patients presenting with chest pain to emergency medicine. Gen Hosp Psychiatry. 2004;26:470–4.

28. Chaturvedi SK, Chandra PS, Channabasavanna S, Anantha N, Reddy B, Sharma S. Levels of anxiety and depression in patients receiving radiotherapy in India. Psychooncology. 1996;5:343–6.

29. Pandey M, Devi N, Thomas BC, Kumar S V, Krishnan R, Ramdas K. Distress overlaps with anxiety and depression in patients with head and neck cancer. Psychooncology. 2007;16:582–6.

30. Kausar R, Ilyas F. A longitudinal study of anxiety in cancer patients before receiving chemotherapy. J Indian Acad Appl Psychol. 2000;26:57–63.

31. Mohanti BK, Kaur J. Living experiences of Indian adult cancer survivors--a brief report. Asian Pacific J Cancer Prev Apjcp. 2015;16:507–12.

32. Imtiaz Ahmad D, Muhammad Waqar A, Memoona K, Ijaz H, Khalid M, Iram H. Depression and anxiety in cancer patients in outpatient department of a tertiary care hospital in Pakistan. Pak J Med Sci [Internet]. 2009;25(5):734–7. Available from: https://pjms.com.pk/issues/octdec109/article/article6.html

33. Jadhav SA, Sukumar S, Kumar G, Bhat SH. Prospective analysis of psychological distress in men being investigated for prostate cancer. Indian J Urol [Internet]. 2010;26:490–3. Available from: https://www.ncbi.nlm.nih.gov/pmc/articles/PMC3034053/

34. Thomas BC, Devi N, Sarita GP, Rita K, Ramdas K, Hussain BM, et al. Reliability and validity of the Malayalam hospital anxiety and depression scale (HADS) in cancer patients. Indian J Med Res. 2005;122:395–9.

35. Mishra SK, Mohapatra PK, Bhattacharya K, Gupta T, Agarwal JP. Prevalence of psychiatric disorder in asymptomatic or minimally symptomatic cancer patients on treatment. J Cancer Res Ther [Internet]. 2006;2:136–9. Available from: http://www.cancerjournal.net/article.asp?issn=0973-1482;year=2006;volume=2;issue=3;spage=136;epage=139;aulast=Mishra

36. Chawla S, Mohanti BK, Rakshak M, Saxena S, Rath GK, Bahadur S. Temporal assessment of quality of life of head and neck cancer patients receiving radical radiotherapy. Qual Life Res. 1999;8:73–8.

37. Alexander PJ, Dinesh N, Vidyasagar MS. Psychiatric morbidity among cancer patients and its relationship with awareness of illness and expectations about treatment outcome. Acta Oncol (Madr). 1993;32:623–6.

38. Chandra PS, Chaturvedi SK, Kumar A, Kumar S, Subbakrishna DK, Channabasavanna SM, et al. Awareness of diagnosis and psychiatric morbidity among cancer patients--a study from South India. J Psychosom Res. 1998;45:257–61.

39. Rashid YA, Ghafoor ZA, Masood N, Mehmood T, Awan S, Ansar T, et al. Psychosocial impact of cancer on adult patients. JPMA - J Pakistan Med Assoc [Internet]. 2012;62:905–9. Available from: http://jpma.org.pk/full_article_text.php?article_id=3671

40. Shankar A, Dracham C, Ghoshal S, Grover S. Prevalence of depression and anxiety disorder in cancer patients: An institutional experience. Indian J Cancer [Internet]. 2016;53:432–4. Available from: http://www.indianjcancer.com/article.asp?issn=0019-509X;year=2016;volume=53;issue=3;spage=432;epage=434;aulast=Shankar

41. Mendonsa RD, Appaya P. Psychiatric morbidity in outpatients of gynecological oncology clinic in a tertiary care hospital. Indian J Psychiatry [Internet]. 2011/01/27. 2010;52(4):327–32. Available from: https://www.ncbi.nlm.nih.gov/pubmed/21267366

42. Chaitanya NC, Garlapati K, Priyanka DR, Soma S, Suskandla U, Boinepally NH. Assessment of Anxiety and Depression in Oral Mucositis Patients Undergoing Cancer Chemoradiotherapy: A Randomized Cross-sectional Study. Indian J Palliat Care [Internet]. 2016;22:446–54. Available from: http://www.jpalliativecare.com/article.asp?issn=0973-1075;year=2016;volume=22;issue=4;spage=446;epage=454;aulast=Chaitanya

43. Nausheen B, Kamal A. Familial social support and depression in breast cancer: an exploratory study on a Pakistani sample. Psychooncology. 2007;16:859–62.

44. Deshpande JD, Phalke DB, Megha B, Tanvi M. Assessment of stress and depression among geriatric inpatients at a tertiary care teaching hospital in rural area. Turk Silahl Kuvvetleri, Koruyucu Hekim Bul. 2014;13:125–32.

45. Afia H, Umar T, Syed Ishtiaq A, Syed Muhammad M, Nida G, Rehan M. Depression in cancer patients attending outpatients department of tertiary care hospitals of Karachi. Ann Abbassi Shaheed Hosp Karachi Med Dent Coll [Internet]. 2013;18(2):101–5. Available from: https://www.annals-ashkmdc.org/pdfs/2013/2/pdf11.pdf

46. Jadoon NA, Munir W, Shahzad MA, Choudhry ZS. Assessment of depression and anxiety in adult cancer outpatients: a cross-sectional study. BMC Cancer [Internet]. 2010;10:594. Available from: https://www.ncbi.nlm.nih.gov/pmc/articles/PMC2988751/

47. Purkayastha D, Venkateswaran C, Nayar K, Unnikrishnan UG. Prevalence of Depression in Breast Cancer Patients and its Association with their Quality of Life: A Cross-sectional Observational Study. Indian J Palliat Care [Internet]. 2017;23:268–73. Available from: https://www.ncbi.nlm.nih.gov/pmc/articles/PMC5545951/

48. Chittem M, Norman P, Harris PR. Relationships between perceived diagnostic disclosure, patient characteristics, psychological distress and illness perceptions in Indian cancer patients. Psychooncology. 2013;22:1375–80.

49. Sutanay B, Sharmistha B, Tanuka M, Das DK. Depression in cancer patients undergoing chemotherapy in a tertiary care hospital of North Bengal, India. Indian J Public Health [Internet]. 2017;61:14–8. Available from: http://www.ijph.in/article.asp?issn=0019-557X;year=2017;volume=61;issue=1;spage=14;epage=18;aulast=Bhattacharyya

50. Khalil A, Faheem M, Fahim A, Innocent H, Mansoor Z, Rizvi S, et al. Prevalence of Depression and Anxiety amongst Cancer Patients in a Hospital Setting: A Cross-Sectional Study. Psychiatry J Print [Internet]. 2016;2016:3964806. Available from: https://www.hindawi.com/journals/psychiatry/2016/3964806/

51. Kulkarni RS, Shinde RL. Depression and Its Associated Factors in Older Indians: A Study Based on Study of Global Aging and Adult Health (SAGE)-2007. J Aging Heal. 2015;27:622–49.

52. Buvneshkumar M, John KR, Logaraj M. A study on prevalence of depression and associated risk factors among elderly in a rural block of Tamil Nadu. Indian J Public Health [Internet]. 2018;62:89–94. Available from: http://www.ijph.in/article.asp?issn=0019-557X;year=2018;volume=62;issue=2;spage=89;epage=94;aulast=Buvneshkumar

53. De S. Subjective assessment of quality of sleep in chronic obstructive pulmonary disease patient and its relationship with associated depression. Lung India [Internet]. 2012;29:332–5. Available from: https://www.ncbi.nlm.nih.gov/pmc/articles/PMC3519018/

54. Misra S, Kundu S, Majumder D, Kundu S, Ghoshal AG, Mitra R. A Study of Depression in Adult Patients with Bronchial Asthma Presenting to a Tertiary Care Hospital in Eastern India. Indian J Chest Dis Allied Sci. 2015;57:87–90.

55. Agarwal A, Batra S, Prasad R, Verma A, Jilani AQ, Kant S. A study on the prevalence of depression and the severity of depression in patients of chronic obstructive pulmonary disease in a semi-urban Indian population. Monaldi Arch Chest Dis. 2018;88:902.

56. Godil A, Mallick MSA, Adam AM, Haq A, Khetpal A, Afzal R, et al. Prevalence and Severity of Depression in a Pakistani Population with at least One Major Chronic Disease. J Clin Diagnostic Res JCDR [Internet]. 2017;11:OC05-OC10. Available from: https://www.ncbi.nlm.nih.gov/pmc/articles/PMC5620817/

57. Khan MA, Sultan SM, Nazli R, Akhtar T, Khan MA, Sher N, et al. Depression among patients with type-II diabetes mellitus. Jcpsp, J Coll Physicians Surg - Pakistan. 2014;24:770–1.

58. Chaudhary R, Kumar P, Chopra A, Chabbra S, Singh P. Comparative Study of Psychiatric Manifestations among Type I and Type II Diabetic Patients. Indian J Psychol Med [Internet]. 2017;39:342–6. Available from: https://www.ncbi.nlm.nih.gov/pmc/articles/PMC5461847/

59. Chaudhry R, Mishra P, Mishra J, Parminder S, Mishra BP. Psychiatric morbidity among diabetic patients: A hospital-based study. Ind Psychiatry J [Internet]. 2010;19:47–9. Available from: https://www.ncbi.nlm.nih.gov/pmc/articles/PMC3105558/

60. Rauf S, Rehman Z, Abrar K. Frequency of psyhiartic morbidity amongst patients with diabetes mellitus in a medical outpatient. A J Army Med Dent Corps. 2005;1.

61. SIDDIQUE I, AHMAD DOGAR I, MALIK S, HAIDER N, AFZAL S, ALI CHEEMA M, et al. Frequency of Depression in Patients With Diabetes Mellitus Type Ii From District Headquarter Hospital, Pakistan. J Pakistan Psychiatr Soc [Internet]. 2014;11(1):31–3. Available from: http://ezproxy.spu.edu/login?url=http://search.ebscohost.com/login.aspx?direct=true&AuthType=ip&db=aph&AN=98502719&site=ehost-live

62. Ali N, Jyotsna VP, Kumar N, Mani K. Prevalence of depression among type 2 diabetes compared to healthy non diabetic controls. J Assoc Physicians India. 2013;61:619–21.

63. Das R, Singh O, Thakurta RG, Khandakar MR, Ali SN, Mallick AK, et al. Prevalence of Depression in Patients with Type II Diabetes Mellitus and its Impact on Quality of Life. Indian J Psychol Med [Internet]. 2013;35:284–9. Available from: https://www.ncbi.nlm.nih.gov/pmc/articles/PMC3821207/

64. Iype T, Shaji SK, Balakrishnan A, Charles D, Varghese AA, Antony TP. Cognition in type 2 diabetes: Association with vascular risk factors, complications of diabetes and depression. Ann Indian Acad Neurol [Internet]. 2009;12:25–7. Available from: http://www.annalsofian.org/article.asp?issn=0972-2327;year=2009;volume=12;issue=1;spage=25;epage=27;aulast=Iype

65. Jain A, Sharma R, Choudhary PK, Yadav N, Jain G, Maanju M. Study of fatigue, depression, and associated factors in type 2 diabetes mellitus in industrial workers. Ind Psychiatry J [Internet]. 2015;24:179–84. Available from: https://www.ncbi.nlm.nih.gov/pmc/articles/PMC4866347/

66. Bahety P, Agarwal G, Khandelwal D, Dutta D, Kalra S, Taparia P, et al. Occurrence and Predictors of Depression and Poor Quality of Life among Patients with Type-2 Diabetes: A Northern India Perspective. Indian J Endocrinol Metab [Internet]. 2017;21:564–9. Available from: https://www.ncbi.nlm.nih.gov/pmc/articles/PMC5477445/

67. Madhu M, Abish A, Anu K, Jophin RI, Kiran AM, Vijayakumar K. Predictors of depression among patients with diabetes mellitus in Southern India. Asian J Psychiatr. 2013;6:313–7.

68. Thour A, Das S, Sehrawat T, Gupta Y. Depression among patients with diabetes mellitus in North India evaluated using patient health questionnaire-9. Indian J Endocrinol Metab [Internet]. 2015;19:252–5. Available from: https://www.ncbi.nlm.nih.gov/pmc/articles/PMC4319265/

69. Mir K, Mir K, Malik I, Shehzadi A. Prevalence of Co-Morbid Depression in Diabetic Population. J Ayub Med Coll Abbottabad JAMC. 2015;27:99–101.

70. Arshad AR, Alvi KY. Frequency of depression in type 2 diabetes mellitus and an analysis of predictive factors. JPMA - J Pakistan Med Assoc [Internet]. 2016;66:425–9. Available from: http://jpma.org.pk/full_article_text.php?article_id=7694

71. Khullar S, Dhillon H, Kaur G, Sharma R, Mehta K, Aggarwal R, et al. The Prevalence and Predictors of Depression in Type 2 Diabetic Population of Punjab. Community Ment Health J. 2016;52:479–83.

72. Islam SM, Ferrari U, Seissler J, Niessen L, Lechner A. Association between depression and diabetes amongst adults in Bangladesh: a hospital based case-control study. J Glob Health [Internet]. 2015;5:20406. Available from: https://www.ncbi.nlm.nih.gov/pmc/articles/PMC4672835/

73. Singh H, Raju MS, Dubey V, Kurrey R, Bansal S, Malik M. A study of sociodemographic clinical and glycemic control factors associated with co-morbid depression in type 2 diabetes mellitus. Ind Psychiatry J [Internet]. 2014;23:134–42. Available from: https://www.ncbi.nlm.nih.gov/pmc/articles/PMC4361976/

74. Rauf U, Ali U. GENDER-BASED DIFFERENCES IN THE LEVEL OF DEPRESSION AMONG PATIENTS WITH TYPE 2 DIABETES. 2016;6–10.

75. Mathew CS, Dominic M, Isaac R, Jacob JJ. Prevalence of depression in consecutive patients with type 2 diabetes mellitus of 5-year duration and its impact on glycemic control. Indian J Endocrinol Metab [Internet]. 2012;16:764–8. Available from: https://www.ncbi.nlm.nih.gov/pmc/articles/PMC3475901/

76. Rajput R, Gehlawat P, Gehlan D, Gupta R, Rajput M. Prevalence and predictors of depression and anxiety in patients of diabetes mellitus in a tertiary care center. Indian J Endocrinol Metab [Internet]. 2016;20(6):746. Available from: http://www.ijem.in/text.asp?2016/20/6/746/192924

77. Weaver LJ, Madhu S V. Type 2 Diabetes and Anxiety Symptoms Among Women in New Delhi, India. Am J Public Health. 2015;105:2335–40.

78. R BYS. Correlates of anxiety and depression among patients with type 2 diabetes mellitus. Indian J Endocrinol Metab 2011 Jul; 15(Suppl 1) S50-4.

79. Ingle VK, Pandey I, Singh AR, Pakhare A, Kumar S. Screening of Patients with Chronic Medical Disorders in the Outpatient Department for Depression Using Handheld Computers as Interface and Patient Health Questionnaire-9 as a Tool. Int J Appl Basic Med Res [Internet]. 2017;7:129–33. Available from: https://www.ncbi.nlm.nih.gov/pmc/articles/PMC5441262/

80. Perveen S, Otho MS, Siddiqi MN, Hatcher J, Rafique G. Association of depression with newly diagnosed type 2 diabetes among adults aged between 25 to 60 years in Karachi, Pakistan. Diabetol Metab Syndr. 2010;2:17.

81. Roy T, Lloyd CE, Parvin M, Mohiuddin KG, Rahman M. Prevalence of co-morbid depression in out-patients with type 2 diabetes mellitus in Bangladesh. BMC Psychiatry. 2012;12:123.

82. Azad N, Gondal M, Abbas N, Shahid A. Frequency of depression and anxiety in patients attending a diabetes clinic. J Ayub Med Coll Abbottabad JAMC. 2014;26:323–7.

83. Gehlawat P, Gupta R, Rajput R, Gahlan D, Gehlawat VK. Diabetes with comorbid depression: role of SSRI in better glycemic control. Asian J Psychiatr. 2013;6:364–8.

84. Farhan A, Ayub AR, Zafar J. Depression In Type-2 Diabetic Patients Presenting To A Tertiary Care Hospital In Pakistan. J Ayub Med Coll Abbottabad JAMC. 2017;29:262–5.

85. Mushtaque A, Gulati R, Hossain MM, Azmi SA. Prevalence of depression in patients of type 2 diabetes mellitus: A cross sectional study in a tertiary care centre. Diabetes Metab Syndr. 2016;10:238–41.

86. Zahid N, Asghar S, Claussen B, Hussain A. Depression and diabetes in a rural community in Pakistan. Diabetes Res Clin Pract. 2008;79(1):124–7.

87. Bajaj S, Agarwal SK, Varma A, Singh VK. Association of depression and its relation with complications in newly diagnosed type 2 diabetes. Indian J Endocrinol Metab [Internet]. 2012;16:759–63. Available from: https://www.ncbi.nlm.nih.gov/pmc/articles/PMC3475900/

88. Natasha K, Hussain A, Khan AK. Prevalence of depression among subjects with and without gestational diabetes mellitus in Bangladesh: a hospital based study. J Diabetes Matabolic Disord [Internet]. 2015;14:64. Available from: https://www.ncbi.nlm.nih.gov/pmc/articles/PMC4517490/

89. Poongothai S, Anjana RM, Pradeepa R, Ganesan A, Unnikrishnan R, Rema M, et al. Association of depression with complications of type 2 diabetes--the Chennai Urban Rural Epidemiology Study (CURES- 102). J Assoc Physicians India. 2011;59:644–8.

90. Raval A, Dhanaraj E, Bhansali A, Grover S, Tiwari P, Amit R, et al. Prevalence and determinants of depression in type 2 diabetes patients in a tertiary care centre. Indian J Med Res. 2010;132:195–200.

91. Lloyd CE, Nouwen A, Sartorius N, Ahmed HU, Alvarez A, Bahendeka S, et al. Prevalence and correlates of depressive disorders in people with Type 2 diabetes: results from the International Prevalence and Treatment of Diabetes and Depression (INTERPRET-DD) study, a collaborative study carried out in 14 countries. Diabet Med. 2018;35:760–9.

92. Asghar S, Hussain A, Ali SM, Khan AK, Magnusson A. Prevalence of depression and diabetes: a population-based study from rural Bangladesh.[Erratum appears in Diabet Med. 2016 Feb;33(2):271; PMID: 26775826]. Diabet Med. 2007;24:872–7.

93. Khuwaja AK, Lalani S, Dhanani R, Azam IS, Rafique G, White F. Anxiety and depression among outpatients with type 2 diabetes: A multi-centre study of prevalence and associated factors. Diabetol Metab Syndr [Internet]. 2010;2(1):72. Available from: http://www.dmsjournal.com/content/2/1/72

94. Zuberi SI, Syed EU, Bhatti JA. Association of depression with treatment outcomes in Type 2 Diabetes Mellitus: a cross-sectional study from Karachi, Pakistan. BMC Psychiatry. 2011;11:27.

95. Siddiqui S, Jha S, Waghdhare S, Agarwal NB, Singh K. Prevalence of depression in patients with type 2 diabetes attending an outpatient clinic in India. Postgrad Med J. 2014;90:552–6.

96. Joseph N, Unnikrishnan B, Raghavendra Babu YP, Kotian MS, Nelliyanil M. Proportion of depression and its determinants among type 2 diabetes mellitus patients in various tertiary care hospitals in Mangalore city of South India. Indian J Endocrinol Metab [Internet]. 2013;17:681–8. Available from: https://www.ncbi.nlm.nih.gov/pmc/articles/PMC3743370/
